# Dissociating the Role of Dorsolateral Prefrontal Cortex and Ventrolateral Prefrontal Cortex in Cognitive Control in Depression: A Combined HD-tDCS and fNIRS Study

**DOI:** 10.1007/s10548-025-01157-4

**Published:** 2025-11-26

**Authors:** Ana Hernández-Sauret, Gonzalo Garcia-Castro, Diego Emilia Redolar-Ripoll

**Affiliations:** 1 Cognitive Neurolab, Faculty of Health Sciences, Universitat Oberta de Catalunya (UOC), Rambla del Poblenou 156, Barcelona, Spain; 2 NeuroDevelopment and Comparative Cognition, Institut de Recerca Sant Joan de Déu (IRSJD), Calle Santa Rosa 39-57, Esplugues de Llobregat, Spain

**Keywords:** Depression, Cognitive control, HD-tDCS, fNIRS, Non-invasive brain stimulation

## Abstract

Major depressive disorder (MDD) is marked by cognitive and affective dysfunctions associated with altered prefrontal cortical activity. While high-definition transcranial direct current stimulation (HD-tDCS) shows promise in modulating these deficits, little is known about the differential effects of targeting specific prefrontal subregions. This study investigated whether HD-tDCS over the dorsolateral (DLPFC) or ventrolateral (VLPFC) prefrontal cortex produces distinct behavioural and neurophysiological effects in patients with MDD, focusing on cognitive control, mood, and functional brain connectivity. Twenty-six patients with MDD received ten sessions of HD-tDCS over the left DLPFC, left VLPFC, or sham stimulation. Assessments were performed pre-intervention, post-intervention, and at one-month follow-up. Measures included the Beck Depression Inventory (BDI), World Health Organization Quality of Life – BREF (WHOQOL-BREF), and performance on cognitive tasks. A subset underwent resting-state functional near-infrared spectroscopy (fNIRS) to assess changes in prefrontal connectivity. DLPFC stimulation led to early and sustained improvements in depressive symptoms, executive function (e.g., Trail Making Test, Wisconsin Card Sorting Task), and quality of life domains. VLPFC stimulation produced delayed improvements, particularly in inhibitory control (e.g., Attention Network Test). fNIRS revealed no significant within-group changes in global connectivity, but at follow-up, the DLPFC group showed greater prefrontal connectivity than both VLPFC and sham, suggesting lasting functional reorganization. VLPFC stimulation did not alter global connectivity, possibly reflecting more localized or subcortical effects. HD-tDCS can differentially modulate cognitive and affective processes in MDD. DLPFC stimulation promotes broader, earlier, and more durable effects, while VLPFC stimulation may exert more specific, delayed influences. Functional connectivity measures enhance interpretation of neuromodulatory outcomes in clinical research.

**Supplementary Information:**

The online version contains supplementary material available at 10.1007/s10548-025-01157-4.

## Introduction

Cognitive control refers to the mental processes that enable individuals to regulate their thoughts and actions in alignment with internal goals, particularly in the presence of distraction, conflict, or shifting demands (Brass et al. 2005). According to Miyake et al. (2000), it allows individuals to inhibit automatic responses, flexibly switch between tasks or strategies, maintain and update information in working memory, and monitor outcomes to adapt behaviour. These functions are crucial for learning, decision-making, and everyday problem-solving, as they promote goal-directed behaviour over habitual or stimulus-driven responses.

Rather than being a unitary construct, cognitive control is best conceptualized as a set of distinct yet interrelated components, often described as having a “unity and diversity” structure (Friedman and Miyake 2017). This idea dates back to Teuber (1972), who used the term to describe the diverse but interconnected functions of the frontal lobe.

The prefrontal cortex is central to cognitive control but is functionally heterogeneous. Notably, two regions have been associated with different aspects of cognitive control: the dorsolateral prefrontal cortex (DLPFC) and the ventrolateral prefrontal cortex (VLPFC). According to MacDonald et al. (2000) and Barbey et al. (2013), the DLPFC is primarily involved in top-down regulation, including planning, goal maintenance, and the maintenance and manipulation of information in working memory. In contrast, the VLPFC is thought to contribute to response inhibition and the selection of relevant stimuli under conditions of interference (Aron et al. 2004; Ryman et al. 2019; Cheng et al. 2022). The prefrontal cortex, including the DLPFC and VLPFC, is not a uniform structure. Rather, it exhibits considerable anatomical and microstructural heterogeneity. Different subregions display distinct cytoarchitectonic properties, such as variations in neuronal density, cell type distribution, and columnar organization, as well as laminar patterns of connectivity that influence how they interact with other cortical and subcortical areas (Badre and Nee 2018; Bruno et al. 2022). These structural differences are thought to underpin the functional specialization observed across prefrontal subregions and may help explain their differential involvement in specific components of cognitive control.

Several studies using neuroimaging techniques have attempted to elucidate the functional roles of these regions. For instance, Hampshire et al. (2010) reported that the VLPFC was selectively engaged during inhibition tasks, whereas the DLPFC was more active during tasks requiring the manipulation of information in working memory (Zhang et al. 2013). The precise functions of these regions and their potential overlap remain an open question, particularly in clinical populations where functional networks may be altered.

Major depressive disorder (MDD) is commonly associated with deficits in cognitive control. These manifestations include difficulties in concentration, sustained attention, inhibition of irrelevant information, decision-making, and cognitive flexibility (Snyder 2013; Joormann and Tanovic 2015; Kriesche et al., 2023). These impairments are increasingly understood not merely as secondary consequences of low mood or fatigue but also as fundamental features of the disorder that often persist even during periods of remission (Vanderhasselt et al. 2009; Rock et al. 2014; Ahern et al. 2025). Historically, both research and clinical practice have focused primarily on the affective symptoms of depression, such as sadness, anhedonia, and hopelessness, whereas cognitive dysfunction has received comparatively less attention. However, a growing body of evidence indicates that deficits in cognitive control play a critical role in the maintenance and recurrence of depressive episodes (see Hernández-Sauret et al. 2024 for a review). Impairments in inhibitory control and cognitive flexibility appear central to sustaining maladaptive cognitive processes, such as rumination, and limiting individuals’ capacity to disengage from negative information (De Raedt and Koster 2010). These cognitive deficits also exert considerable impact on daily functioning, affecting occupational performance, academic achievement, and social relationships. Furthermore, they have been associated with poorer treatment outcomes and slower recovery trajectories, emphasizing the importance of directly addressing cognitive symptoms in both research and therapeutic interventions (Bora et al. 2013). Notably, emerging findings suggest that these impairments may be rooted in functional disruptions within the prefrontal cortex, opening a critical avenue for understanding the neural underpinnings of cognitive dysfunction in MDD.

MDD has been consistently linked to disrupted functioning within prefrontal cortical networks, as evidenced by neuroimaging research (Zhu et al. 2018; Lang et al. 2021; Steinmann et al. 2024). In particular, studies have demonstrated reduced activation of the DLPFC during tasks requiring executive functions such as working memory, planning, and goal maintenance (Kaiser et al. 2015). Similarly, the VLPFC has shown altered activation and connectivity patterns in individuals with MDD. A recent study by Keller et al. (2021) using fMRI neurofeedback revealed that patients with depression exhibited diminished activation in the left VLPFC during cognitive reappraisal and demonstrated reduced connectivity with parietal and limbic regions compared to healthy controls. These alterations were associated with reduced use of adaptive emotion regulation strategies and greater symptom severity.

These findings suggest that MDD is characterized by a functional breakdown in prefrontal circuitry, which may underlie both the cognitive and affective symptoms of the disorder. Importantly, such dysfunction is not only present during acute episodes but can also persist during remission, indicating that it may reflect stable trait-like vulnerability. Delineating the distinct contributions of DLPFC and VLPFC circuit-level perturbations to the neurobiological underpinnings of depression is critical for informing the development of mechanistically targeted neuromodulatory interventions. In recent years, this line of research has increasingly pointed toward neuromodulatory approaches, including non-invasive brain stimulation techniques, as promising tools for reinstating prefrontal function and alleviating depressive symptomatology.

Non-invasive brain stimulation (NIBS) techniques have gained increasing attention as tools for modulating dysfunctional neural circuits in patients with psychiatric disorders, particularly MDD. Among these methods, high-definition transcranial direct current stimulation (HD-tDCS) has emerged as a promising and more focal alternative to conventional transcranial direct current stimulation (tDCS). HD-tDCS delivers low-intensity electrical currents through specialized electrode montages, allowing for greater spatial precision in targeting specific cortical regions, such as the DLPFC or VLPFC (Villamar et al. 2013; Jog et al. 2025). This improved focality is especially relevant when attempting to modulate distinct prefrontal subregions that support different aspects of cognitive control.

Despite growing evidence linking prefrontal dysfunction to cognitive impairments in depression, the specific contributions of distinct prefrontal subregions, such as the DLPFC and VLPFC, to different components of cognitive control remain insufficiently understood. Most neuromodulation studies have focused on the DLPFC, overlooking the potential role of the VLPFC in processes such as inhibition and interference control.

To address this gap, the present study investigated whether HD-tDCS over either the DLPFC or the VLPFC produces differential effects on cognitive control performance in individuals with MDD. By comparing behavioural outcomes following stimulation of these two regions, this study sought to clarify their functional contributions and support the development of more targeted therapeutic approaches for cognitive dysfunction in depression. In addition to identifying this functional dissociation, the protocol also examines whether stimulation leads to sustained cognitive improvements over time. Moreover, we investigated how functional connectivity patterns within the prefrontal cortex change as a result of the intervention via functional near-infrared spectroscopy (fNIRS). This multimodal approach allows us to assess both the behavioural and neurophysiological effects of HD-tDCS, contributing to a more comprehensive understanding of its therapeutic potential.

## Methods

### Study Overview and Experimental Design

Twenty-six patients diagnosed with depression were recruited for this study and randomly assigned to one of three independent stimulation conditions: HD-tDCS over the left DLPFC, HD-tDCS over the left VLPFC, or sham stimulation. The participants were blinded to the stimulation conditions. Each participant underwent 10 consecutive sessions of HD-tDCS, which were administered from Monday to Friday over two weeks.

**Fig. 1:**
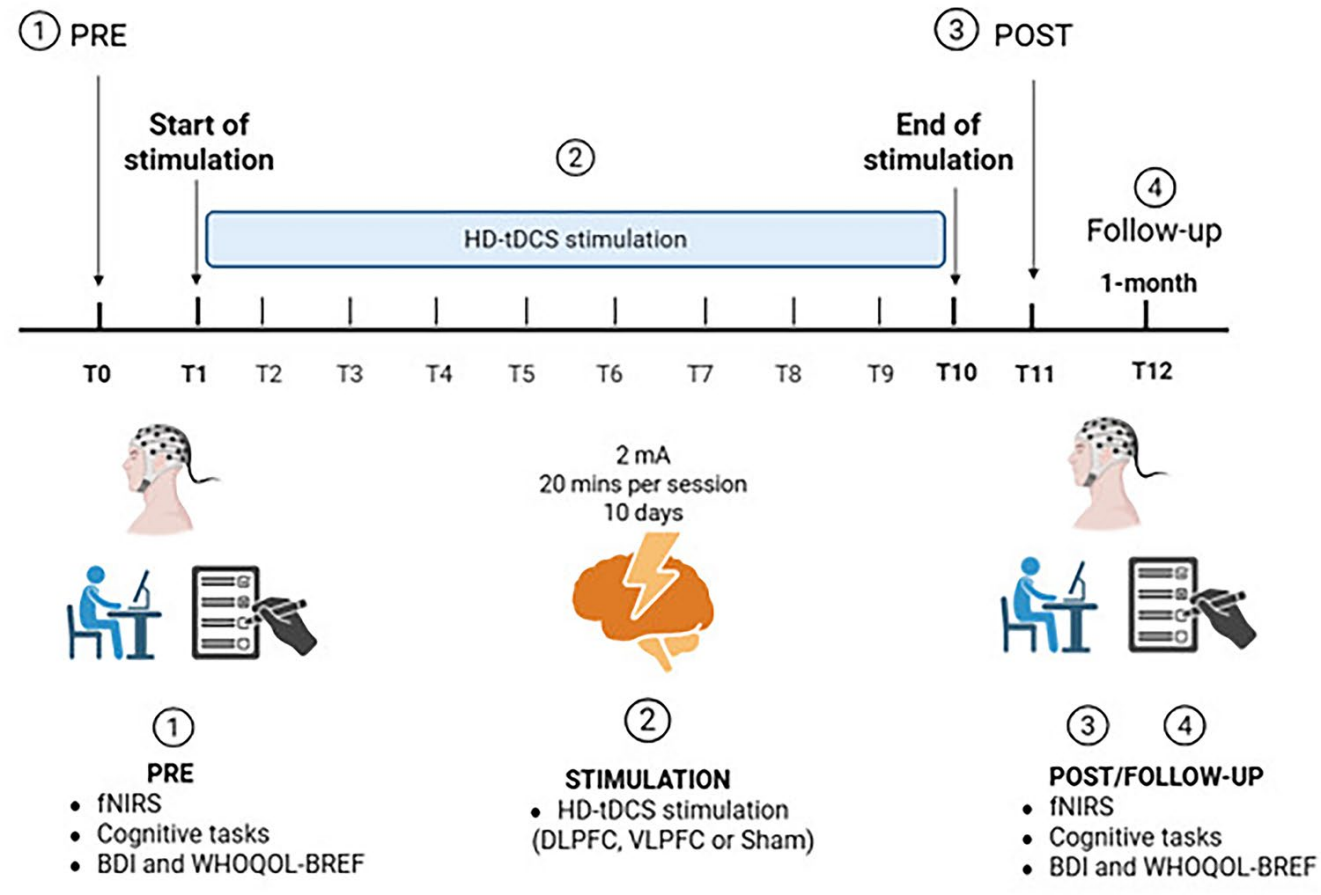
Experimental protocol. Participants underwent baseline assessments (T0): fNIRS, cognitive tasks, and questionnaires (BDI and WHOQOL-BREF), followed by 10 consecutive weekday sessions of HD-tDCS targeting the DLPFC, VLPFC, or sham stimulation (T1–T10). Post-intervention assessments were conducted immediately after stimulation (T11) and at one-month follow-up (T12). Figure created with BioRender.com

One day before the first stimulation session (pre), the participants completed a baseline assessment, including the Beck Depression Inventory (BDI), the World Health Organization Quality of Life – BREF (WHOQOL-BREF), and cognitive tasks. In addition, resting-state brain activity was recorded with fNIRS. These same assessments were repeated 48 h after the 10th session (post) and again one month after the end of the stimulation protocol (follow-up). See Fig. 1.

All experimental sessions were conducted between 8:00 AM and 7:00 PM according to participant availability, with each participant tested at the same time of day throughout the study to minimize circadian influences.

Participants were systematically monitored for safety and tolerability throughout the intervention. Before each stimulation session, they were asked whether they had experienced any adverse effects since the previous day. At the end of the session, they were again asked how they felt and whether they had noticed any side effects (e.g., headache, itching, skin redness). This procedure was repeated daily for all participants.

### Participants

Twenty-six patients diagnosed with depression were recruited for this study. The groups were matched for age and sex, and no significant differences were found in the baseline clinical or quality of life measures. Depressive symptomatology, as measured by the BDI, was comparable across groups. Similarly, no significant group differences were detected in the WHOQOL-BREF domains, including physical health, psychological well-being, interpersonal relationships, the environment, or the total quality of life score (Table 1). However, one-way ANOVA revealed a significant group effect for depression duration (F(2, 23) = 6.511, *p* = 0.006). Post hoc comparisons indicated that participants in the VLPFC group had a significantly shorter history of depression than did those in both the DLPFC (*p* = 0.015) and Sham (*p* = 0.015) groups, whereas no difference was found between the DLPFC and Sham groups (*p* = 0.977) (Table 1).

Participants with metal implants in the head, a cardiac pacemaker, current pregnancy, or a history of seizures were excluded from the study. None of the participants reported taking any medication that could interact with the effects of tDCS. Psychopharmacological treatment was not changed for at least 2 weeks before HD-tDCS application (Bikson et al. 2017; Kang et al. 2024).

Three participants dropped out of the study before completing the stimulation sessions, one due to insomnia and two for unknown reasons. Additionally, two more participants did not return for the one-month follow-up assessment, despite completing the intervention and post-assessment phases.

**Table 1:** Comparison of demographic and clinical variables at baseline for the DLPFC, VLPFC, and Sham groups

	DLPFC			VLPFC			Sham			Statistics		
	*N*	Mean	SD	*N*	Mean	SD	*N*	Mean	SD	df	F	*p* value
Gender: female/male	7/2			8/2			7/0					
Working status: yes/no	6/3			4/6			5/2					
Education: Secondary/University	2/7			4/6			3/4					
Age, years		38,4	14,3		43,6	11,4		32,4	9,4	2,0	1,5	0,2
Duration of depression, years		4,6	4,1		15,2	11,4		3,9	1,2	2,0	6,5	0,006*
Duration of medication, years		1,0	0,8		2,9	4,0		0,8	1,1	2,0		0,7
Baseline BDI		35,1	13,8		31,3	12,3		29,7	17,0	2,0	0,3	0,7
Baseline WHOQOL-BREF		32,9	12,3		40,2	15,9		47,3	13,4	2,0	2,1	0,1

BDI, Beck Depression Inventory; WHOQOL-BREF, Quality of Life-BREF questionnaire

### Assessment

To evaluate depressive symptoms, each patient completed BDI. This evaluation is a 21-item self-report questionnaire used to assess the severity of depression. Each question presents multiple statements about depressive symptoms, and respondents select the statement that best reflects their feelings over the past two weeks. Answers are scored from 0 to 3, with higher scores indicating more severe depression. The total score helps classify the level of depression (minimal, mild, moderate, or severe) and aids in monitoring symptoms or evaluating treatment progress.

The WHOQOL-BREF was used to assess an individual’s overall quality of life. It includes 26 questions that evaluate four key areas: physical health, psychological health, social relationships, and the environment. The participants rated their experiences from 1 (not at all) to 5 (an extreme account) over the past two weeks, and the responses helped them evaluate overall well-being. Higher scores indicate better quality of life in each domain.

### Behavioral Task

Each participant had the same sequence of cognitive tasks.

Executive function was evaluated through the Trail Making Test (TMT). In Part A, the participant was shown numbered circles scattered across the screen. The task consisted of drawing a line connecting the numbers in ascending order (1 to 2, 2 to 3, and so on) as quickly as possible. This part primarily measures visual attention and processing speed. In Part B, the participant had to alternate between numbers and letters. For example, they connect 1 to A, then A to 2, 2 to B, B to 3, and so on, continuing this alternating sequence. This part tests not only attention and speed but also cognitive flexibility, as the participant must switch between numerical and alphabetical sequences.

The Corsi Block Tapping Test (CBTT) was used to assess working memory. The screen displayed several squares arranged in a random pattern. The computer lit up a sequence of blocks, one by one, in a specific order. The participant’s task was to watch and then replicate the sequence by clicking on the blocks in the same order.

The Wisconsin Card Sorting Task (WCST) was used to assess cognitive flexibility. The participants sorted cards on the screen based on colour, shape, or number, but were not told the sorting rule. They received feedback (correct or incorrect) after each attempt. Periodically, the sorting rule changes without notice, requiring participants to adjust their strategy based on the feedback.

Phonetic fluency involves strategic search and retrieval processes within orthographic or phonological networks and thus requires several higher-order functions. The participants executed the FAS verbal fluency test, which required an individual to produce as many words as possible for 1 min, beginning with the letter F. The same procedure was repeated for the letters A and S. Proper nouns and plurals were not permitted.

Attention was measured with the Attention Network Test (ANT). The participants were shown cues (one or two asterisks) that could predict the location of an upcoming target or provide information about its location. After the cue, the target arrows appeared individually or in an array of five. The participants had to respond by indicating the direction in which the central arrow was pointing.

Inhibition was assessed via the Stroop test, a classic measure of cognitive control and interference resolution. The task included three conditions presented sequentially. First, participants were asked to read aloud a list of color words printed in black ink (e.g., “red,” “blue”), measuring baseline reading speed. Next, they identified the ink color of a series of colored rectangles, assessing the baseline color naming. Finally, in the interference condition, the participants viewed color words printed in incongruent ink colors (e.g., the word “blue” printed in red ink) and were instructed to name the color of the ink, not the word itself. This condition required overriding the automatic tendency to read the word, providing an index of inhibitory control. This was the only paper-based task; all other tasks were administered via computer.

### HD-tDCS Procedure

**Fig. 2:**
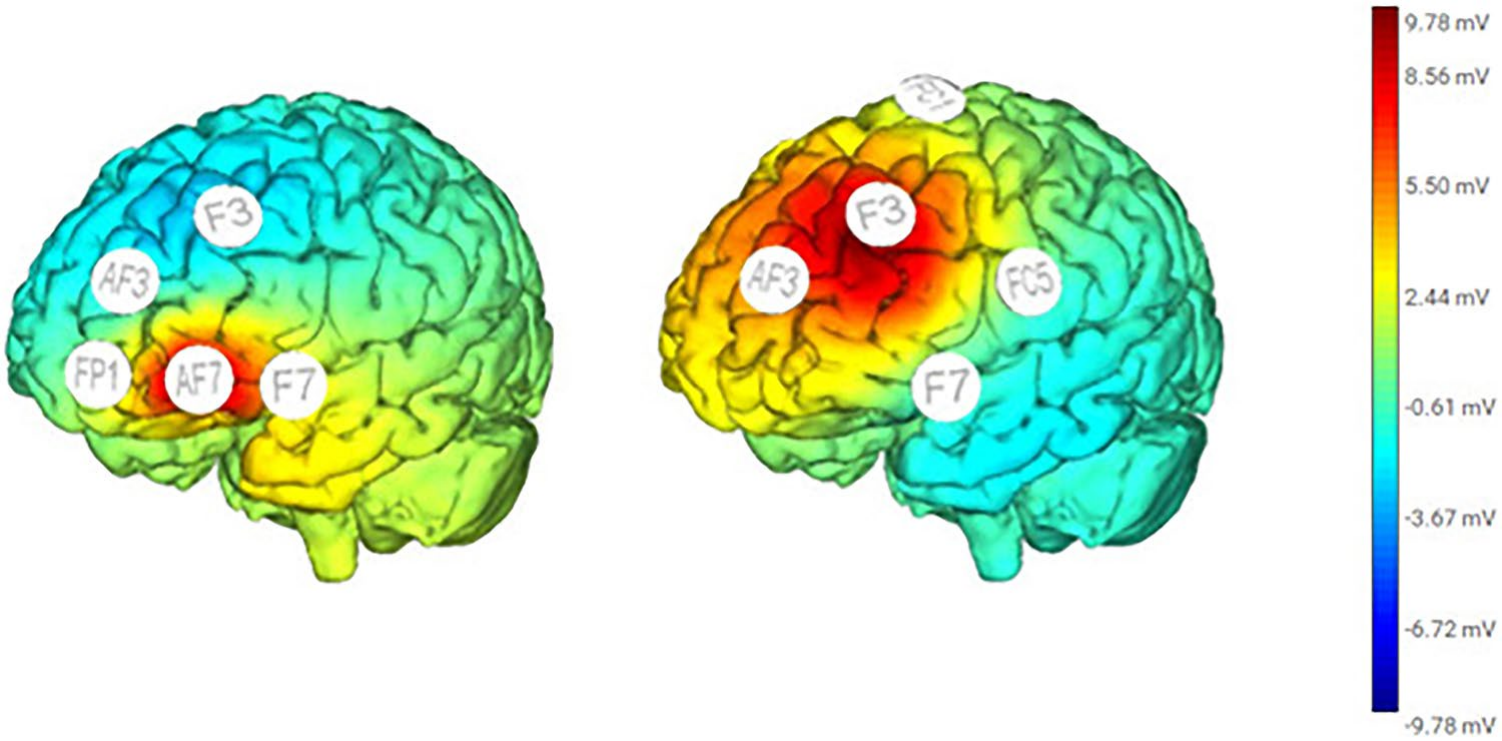
Simulation of current distribution during HD-tDCS over the VLPFC (left) DLPFC (right), generated via NIC2 software from Neuroelectrics. Warmer colours (red/yellow) indicate regions with higher current density (in mA), whereas cooler colours (blue/green) reflect lower intensity. These simulations represent the modelled distribution of electrical stimulation based on specific electrode montages applied over the left hemisphere

The stimulation was administered using Starstim^®^, produced by Neuroelectrics. The HD-tDCS was controlled through Wi-Fi via a computer via Starstim^®^ software. The montages were the 4 × 1 ring set-up, which is a typical HD-tDCS stimulation protocol. There was a central anodal electrode surrounded by 4 return cathodal electrodes. For the DLPFC montage, the anode was placed over the left DLPFC, which was located at F3, based on the 10/20 electroencephalogram system. The four cathodal electrodes were placed at AF3, FC1, FC5, and F7. For the VLPFC montage, the anode was placed over the left VLPFC, which was located at AF7. The return cathodal electrodes were placed at FP1, AF3, F3 and F7 (Fig. 2). For the Sham montage, the anode and return cathodes were placed at the same position as for DLPFC montage. Sham stimulation, a control procedure used to assess responses in the absence of current while keeping participants blind to the condition (Ambrus et al. 2012; Palm et al. 2013; Garnett and den Ouden 2015), involved delivering HD-tDCS current for the first seconds and ramping it down after 30 s. Conductive electrode gel was applied to the scalp at all designated stimulation areas. To ensure that the electrodes were secured in place, a different cap size was used depending on the subject’s head size. Before each session, impedance checks were performed using the Starstim^®^ software. The stimulation delivered was at 2 mA (current density 0,63 mA/cm^2^) for 20 min, with a gradual ramp-up and ramp-down of the current over the initial and last 30 s. The participants were instructed to remain relaxed and still during the procedure. Throughout each session, the administrator monitored impedance levels and recorded any side effects reported by the participants, who were regularly asked about any discomfort. Each session lasted approximately 45 min and was conducted five days a week for two consecutive weeks.

### Cognitive and Affective Statistical Analysis

All the cognitive and affective statistical analyses were conducted using JASP software (version 0.19.1; JASP Team, University of Amsterdam). To assess the effects of the different stimulation protocols over time and between groups, both parametric and nonparametric tests were applied, depending on the data distribution and assumptions.

Normality was evaluated using the Shapiro–Wilk test, and when assumptions of normality and sphericity were met, repeated-measures ANOVAs were performed to analyse within-group changes across the three time points (pre-intervention, post-intervention, and 1-month follow-up). When the sphericity assumption was violated, the Greenhouse–Geisser correction was applied. Post hoc pairwise comparisons were adjusted using Holm’s correction for multiple testing.

When the data did not meet the assumptions for parametric testing, non-parametric alternatives were used. Specifically, the Friedman test was applied for repeated measures, and Wilcoxon signed-rank tests were used for post hoc comparisons, which were also corrected using Holm’s method. Effect sizes such as Kendall’s W were reported for non-parametric results.

Baseline group differences were evaluated using one-way ANOVA for normally distributed variables and the Kruskal–Wallis test for non-parametric variables. All the statistical tests were two-tailed, and a significance threshold of *p* < 0.05 was used for all the analyses.

Concerning the sample size and statistical power, our primary aim was to assess within-group change over time. We therefore conducted post-hoc sensitivity analyses in G*Power 3.1 (repeated-measures ANOVA, within factors; 3 measurements; α = 0.05; 1 − β = 0.80; assumed correlation among repeated measures = 0.50; ε = 1) separately for each group (DLPFC *n* = 9; VLPFC *n* = 10; Sham *n* = 7). The detectable within-group effects were f = 0.465 (ηp² ≈ 0.178) for DLPFC, f = 0.437 (ηp² ≈ 0.160) for VLPFC, and f = 0.546 (ηp² ≈ 0.229) for Sham. Conversion between Cohen’s f and partial eta squared was performed using the standard formula:

$$\:{\eta\:}_{\rho\:}^{2}=\frac{{f}^{2}}{1+{f}^{2}}$$

Observed effect sizes for cognitive outcomes (e.g., Stroop, FAS) generally exceeded these thresholds (see Table 2), indicating adequate sensitivity despite modest group sizes. For outcomes analysed with Friedman tests, we report Kendall’s W, as power analyses for W are not supported in G*Power.

### fNIRS Data Acquisition and Processing

**Fig. 3:**
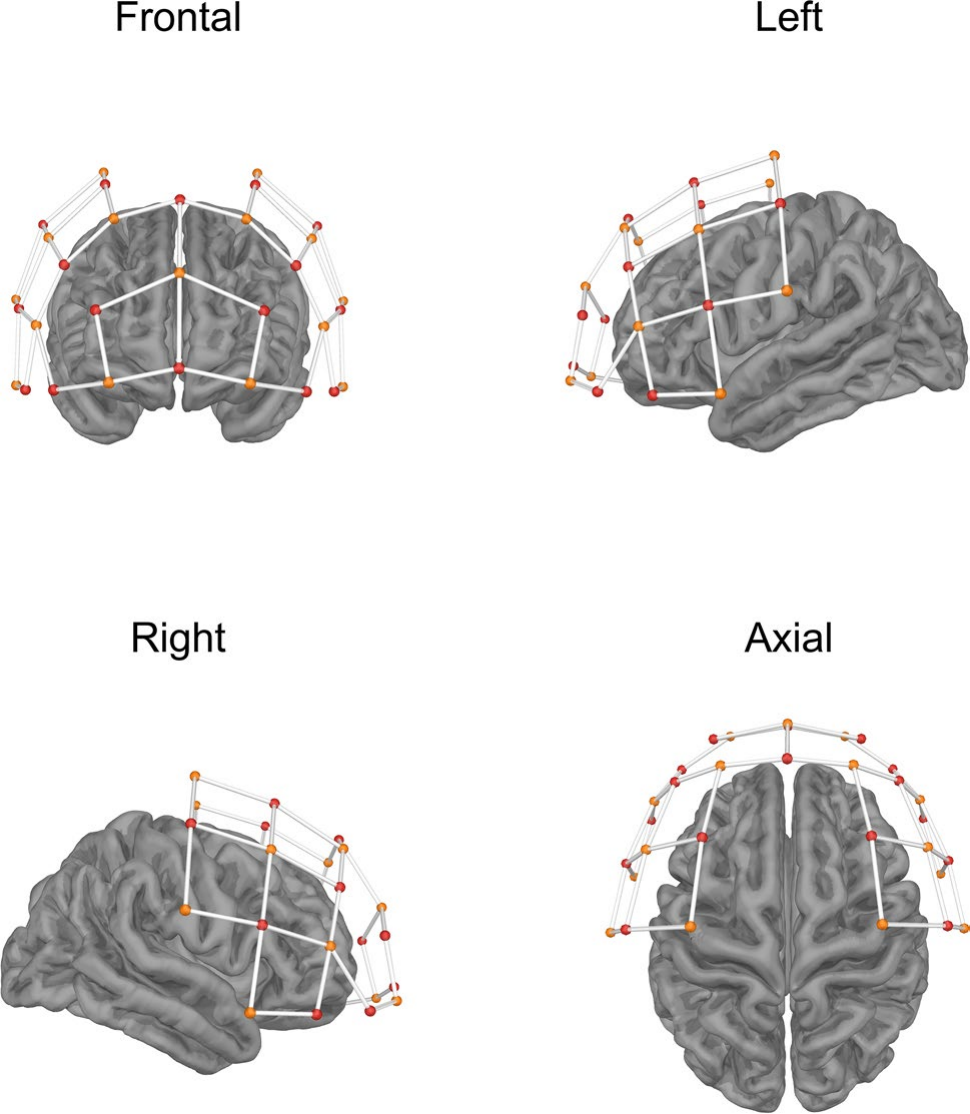
Frontal, left, right, and axial views of the optode layout and source-detector pairs over the frontal cortex. Red and yellow dots represent sources and detectors, respectively, and white lines represent channels

fNIRS to measure changes in the relative concentrations of oxy-hemoglobin (HbO) and deoxyhemoglobin (HbR) in cortical areas of interest. fNIRS analyses were conducted on a subset of 14 participants, including 5 in the DLPFC group, 5 in the VLPFC group, and 4 in the sham group. We used a NIRSport2 instrument (NIRx Medical Technology LLC, NY, USA) with two continuous-waves at 760 nm and 850 nm and a sampling rate of 5.1 Hz. The setup included 16 sources and 16 detectors, which were arranged according to the international 10–20 system to cover the frontal cortex (Fig. 3). Source-detector pairs (i.e., channels) were separated by an average of 3.5 cm, SD = 0.39 cm, range = 2.98–4.09). Additionally, eight short-distance channels (separated by an average of 0.76 cm, SD = 0.03, range = 0.71–0.81) were placed at Fpz, AF3, F7, FC1, C3, F4, FC2, and FC6 to capture extra-cerebral hemodynamic signals, which were later regressed out from the cortical data.

NIRS data were pre-processed in the Python 3.12.0 programming environment using the MNE-Python (Gramfort et al. 2013) and MNE-NIRS (Luke et al. 2021) toolboxes. First, raw light intensity data were transformed to optical density. Motion artifacts were attenuated using the temporal derivative distribution repair (TDDR) (Fishburn et al. 2019). To remove signal fluctuations originated in extra-cerebral tissue from the signal, we applied short-separation channel regression (Fabbri et al. 2004). Signal quality was then assessed on a channel-wise basis using the scalp coupling index (SCI) (Pollonini et al. 2014): channels with less than 0.7 SCI were marked as bad and later interpolated using spherical spline interpolation (Perrin et al. 1989). We then inspected each dataset manually, annotating time segments containing high-amplitude noise or remaining motion artifacts. Optical densities were then converted into changes in HbO and HbR concentrations using the modified Beer–Lambert law with a partial pathlength factor (PPF) of 6.0 for both wavelengths. Finally, the signal to noise ratio was improved by using the negative correlation enhancement (Cui et al. 2010). Only long-separation channels were retained for analysis. To attenuate physiological artifacts related to cardiac pulsation (1–1.5 Hz), respiration (0.2–0.5 Hz), and low-frequency blood pressure oscillations (Mayer waves, ~ 0.1 Hz), a band-pass filter between 0.01 and 0.20 Hz was applied, with a transition width of 0.01 Hz for the lower bound and 0.2 Hz for the upper bound.

### Resting State Functional Connectivity Measures

**Fig. 4:**
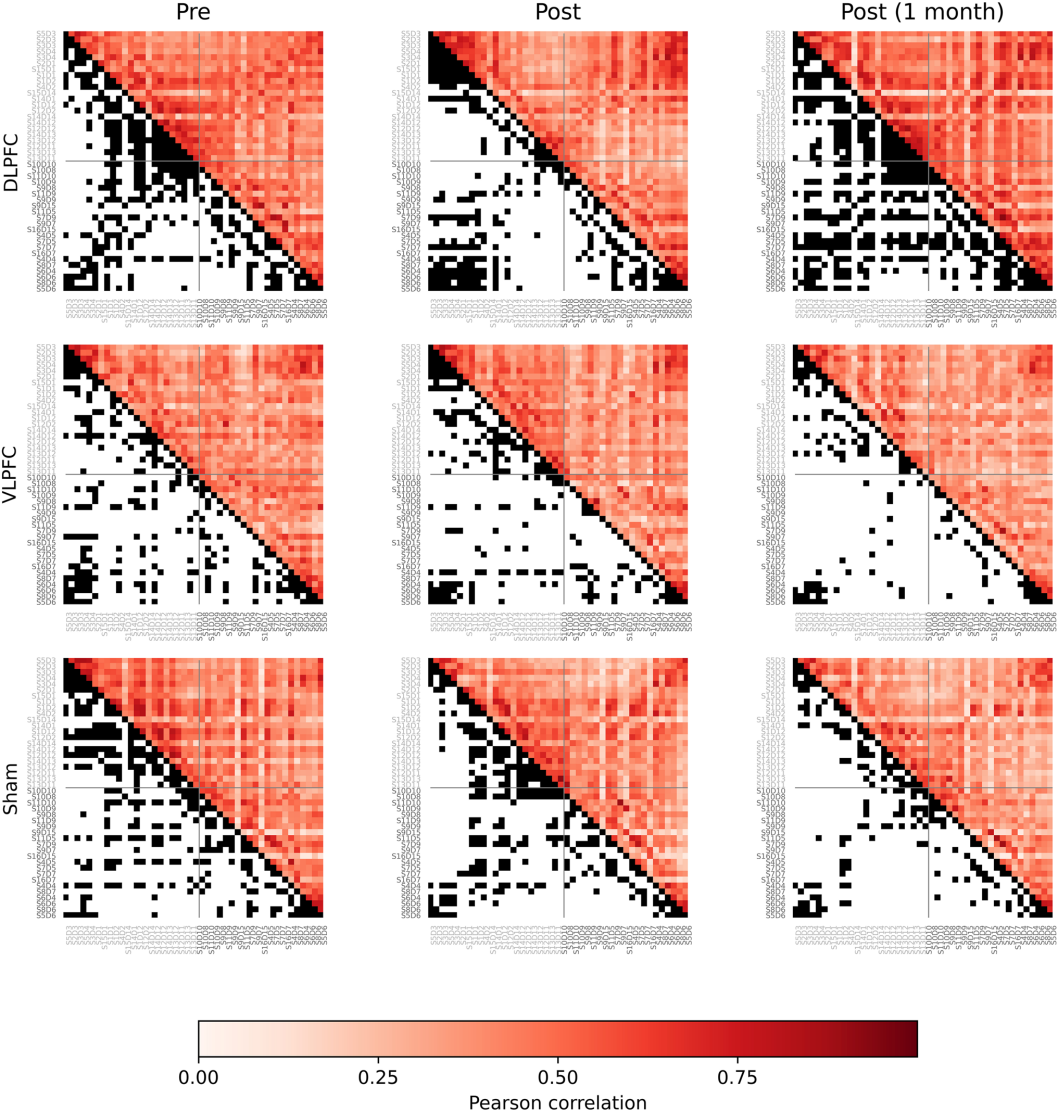
Average functional connectivity matrices for participants in groups DLPFC, VLPFC, and Sham, across the three testing sessions. The upper triangle of each matrix shows the Pearson correlation coefficient between each pair of channels. The lower triangle of each matrix shows the binarized values after applying the threshold. Black dots in the binarized matrix indicate Pearson correlation coefficient values equal or higher than the threshold. Channels were ordered from left (black) to right (grey) and from anterior to posterior

Each recording was segmented into 30-second-long epochs, with a five-second overlap between consecutive epochs. Epochs that overlapped with the previously annotated segments containing high-amplitude noise or motion artifacts or for which the HbO signal reached a saturation value (10 µM) were excluded from further analyses. We then calculated the undirected functional connectivity matrix of each dataset by computing the pairwise absolute value of the Pearson correlation coefficient between the HbO time series of the 44 channels. The resulting matrix was binarized by applying a threshold of 0.5 so that values equal to or above the threshold were assigned a value of one and zero otherwise. Figure 4 shows the average weighted and binarized functional connectivity matrix for each group in each testing session. We then used the Python implementation of the Brain Connectivity Toolbox (Rubinov et al. 2009; Rubinov and Sporns 2010) to calculate the connectivity degree of each channel. This graph-theoretical measure, indicates the number of functional connections a given node (channel) has with the rest of the network, providing a measure of its centrality and potential influence within the brain network. (Rubinov and Sporns 2010).

### fNIRS Data Analysis

We modelled the data using a generalized multilevel Bayesian model, specifying a Poisson probabilistic distribution to model the response variable. As fixed effects, we included (1) *Degree* as the response variable, (2) *Group* (DLPFC, VLPFC, Sham), *Session* (Pre, Post, Post (1 month), and their two-way interaction^1^.

We specified random intercepts and random slopes for the *Session* predictors for both random effects. For each channel, we additionally specified random slopes the *Group* predictor and its interaction with *Session*. We specified weakly informative priors for the parameters in the model (see Eq. 1 for a formal representation of the statistical model). We implemented the model in the R 4.5.0 programming environment using brms (Bürkner 2017), an R interface to the probabilistic programming language Stan (Carpenter et al. 2017). Model parameters were estimated by running eight Monte-Carlo Markov Chains (MCMC) with 2,000 iterations each (1,000 of which were warm-up iterations).

1 $$\:\begin{array}{rr}&\:\text{Degree\:for\:channel}\hspace{0.25em}i\hspace{0.25em}\text{and\:participant}\hspace{0.25em}j:\\\:y&\:\sim\:\text{Poisson}\left(\alpha\:\right)\\\:&\:\text{Distributional\:parameters:}\\\:\text{l}\text{o}\text{g}\left(\alpha\:\right)&\:=\left({\beta\:}_{0}+{u}_{0i}+{w}_{0j}\right)+\left({\beta\:}_{1}+{u}_{1i}\right)\cdot\:{\text{Group}}_{ij}+({\beta\:}_{2}+\\\:&\:{u}_{2i}+{w}_{1j})\cdot\:{\text{Session}}_{ij}+\left({\beta\:}_{3}+{u}_{3i}\right)\cdot\:{\text{Group}}_{ij}\cdot\:{\text{Session}}_{ij}\\\:&\:\text{Priors:}\\\:{\beta\:}_{0,1,2,3}&\:\sim\:\mathcal{N}\left(0,2.5\right)\\\:{u}_{0,1,2,3}&\:\sim\:(0,{\sigma\:}_{{u}_{0,1,2,3}})\\\:{w}_{0,1,2}&\:\sim\:(0,{\sigma\:}_{{w}_{0,1,2}})\\\:{\sigma\:}_{{u}_{0,1,2,3}},{\sigma\:}_{{u}_{0,1,2}}&\:\sim\:\text{e}\text{x}\text{p}\left(2\right)\end{array}\:\:\:\:$$

We performed statistical inference and hypothesis testing on the average marginal posterior predictions of the model. After estimating the model’s posterior distribution, we generated 1,000 mean posterior predictions for each combination of levels of Group and Session and then calculated the pairwise difference between the draws of each combination of levels (Δ). This resulted in a posterior distribution of differences for each pairwise comparison. We then calculated, for each distribution, the 89% highest density interval (HDI) around the median, which encompasses the smallest interval that contains 89% of the values of the distribution. This interval contains the true value of the median of the distribution with 89% probability, given the observed data. Following Kruschke and Liddell (2018), we finally define a region of practical equivalence (ROPE) around zero. This region contains the values that were considered equivalent to zero. We specified the lower and upper bounds of the rope as zero plus/minus one-tenth of the standard deviation of the response variable (connectivity degree) (SD = 9.96), ROPE = [−0.996, 0.996] (Kruschke and Liddell 2018; McElreath 2018). We compared the 89% HDI of each distribution of differences against the ROPE by calculating the proportion of overlap between both. This value provides an estimated probability of each of the pairwise differences being equivalent to zero (ROPE). If ROPE < 0.025, the hypothesis that the difference is zero is rejected. If ROPE > 0.975, the hypothesis that the difference is zero is supported. Intermediate values, or situations in which the 89% HDI crosses both lower and upper bounds of the ROPE, are interpreted as inconclusive.

## Results

This section presents the results of the analyses conducted to examine the effects of the different stimulation protocols on depressive symptoms, quality of life, and cognitive performance across time. First, baseline comparability between groups was verified to ensure that there were no preexisting differences in mood, quality of life, or cognitive function.

### Adverse Effects and Tolerability

Across groups, HD-tDCS was generally safe and well tolerated, with only mild and transient adverse effects reported. In the DLPFC group, five participants experienced headache immediately after stimulation, which resolved spontaneously within a short period. In the VLPFC group, four participants also reported transient headaches, and six presented with temporary skin redness at the site of the anode. In the sham group, one participant reported a mild headache after a single session. Importantly, none of these effects were bothersome or disabling for participants, and no adverse event interfered with task performance or completion of the intervention. No serious adverse events were observed, and all participants were able to complete the intervention protocol. These findings are consistent with previous reports indicating that HD-tDCS is a safe and well-tolerated intervention in clinical populations (Ngan et al. 2022; Lu et al. 2025; Wan et al. 2025).

### Questionnaire

The comparison of the baseline measures in BDI and Quality of Life questionnaire using ANOVA or Kruskal-Wallis for non-parametric data, showed no statistically significant difference for any variable. Given this result, the three groups were considered to have equivalent performance levels at baseline.

1. BDI

In the DLPFC group, a repeated-measures ANOVA revealed a significant effect of time (*F(2*,*14)* = 13.38, *p* < 0.001, ηp²=0.657). Post hoc comparisons with Holm’s correction revealed significant reductions in depressive symptoms from pre- to post-intervention (*p* = 0.007) and from pre- to 1-month follow-up (*p* = 0.01), with no difference between post and follow-up (*p* = 0.417).

In the VLPFC group, a Friedman test indicated a significant effect of time (χ^2^ (2) = 7.20, *p* = 0.027, Kendall’s W = 0.36). A significant improvement was found from pre- to follow-up (*p* = 0.015), whereas comparisons between pre and post, and post and follow-up, were not significant.

**Fig. 5:**
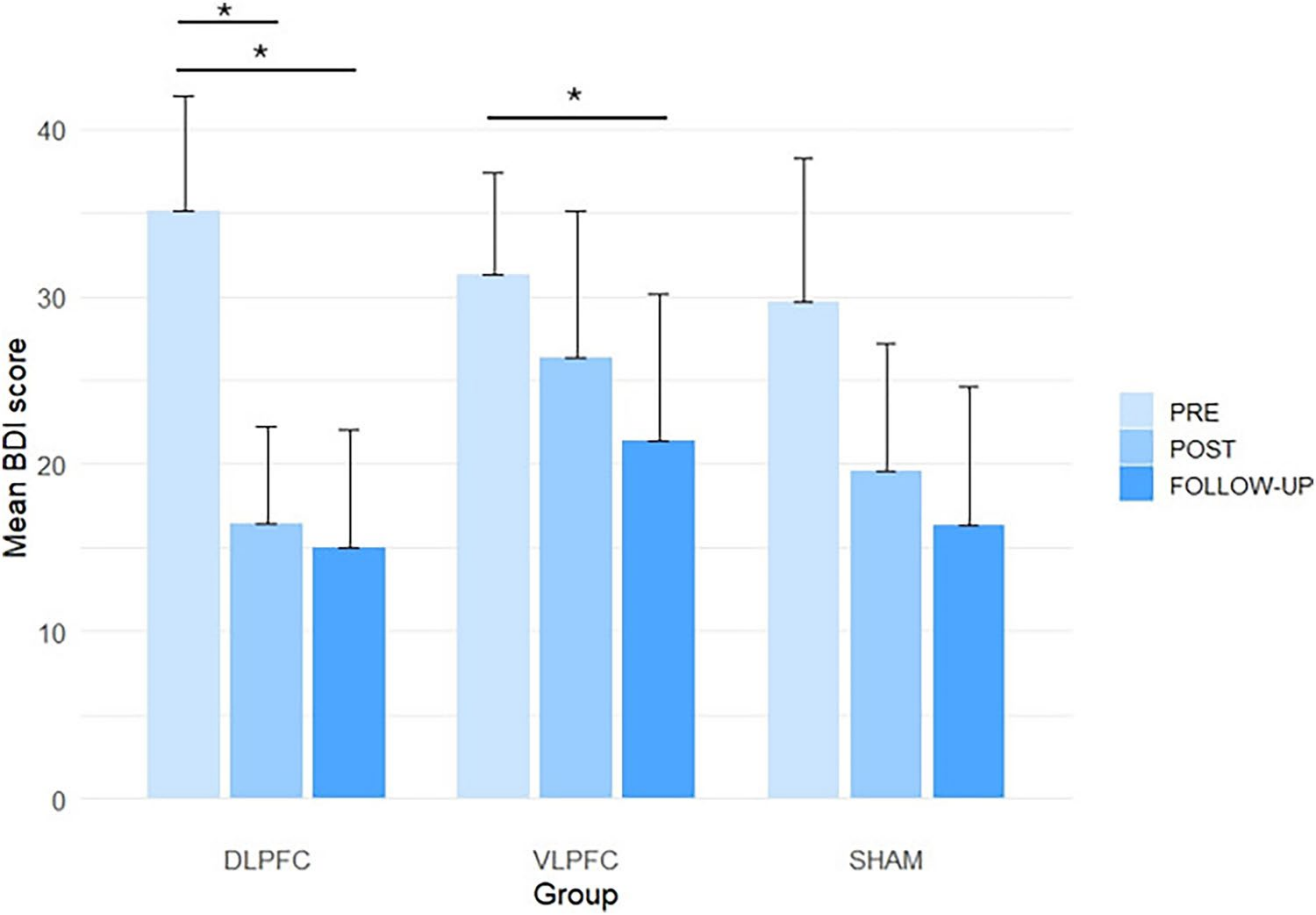
Mean BDI scores at baseline (PRE), after the intervention (POST), and at the one-month follow-up (Follow-up) for each experimental group (DLPFC, VLPFC, SHAM). Error bars represent the standard error of the mean (SD). Depressive symptoms improved significantly (* *P* < 0.05) in DLPFC and VLPFC group

In the Sham group, a repeated-measures ANOVA revealed a significant effect of time (*F(2*,*10)* = 4.96, *p* = 0.032, ηp²=0.498), although no post hoc comparisons reached significance (*p* > 0.05). (Fig. 5)

2. WHOQOL-Bref

In the DLPFC group, using ANOVA, a significant effect of time was observed on the total score (F(2,14) = 5.16, *p* = 0.021, ηp²=0.42), with pairwise comparisons showing marginally significant improvements from pre- to post-intervention (*p* = 0.052) and from pre- to follow-up (*p* = 0.056), indicating a marked trend toward improvement over time. The physical health domain also showed a significant effect (F(2,14) = 9.46, *p* = 0.003, ηp²=0.575), with improvements from pre- to follow-up (*p* = 0.004). The psychological domain revealed a strong effect of time (F(2,14) = 12.61, *p* < 0.001), with significant gains from pre to both post (*p* = 0.003) and follow-up (*p* = 0.009). No changes were observed in interpersonal relationships or the environment.

The VLPFC group showed no significant changes over time in the total score (F(2,18) = 0.15, *p* = 0.86, ηp²= 0.016) or any domain after the ANOVA repeated measured was applied.

In the Sham group, the Friedman test revealed no significant effect for the total score (χ²2 = 9.0, *p* = 0.638, W = 0.075), but a significant improvement was observed in physical health (χ²2 = 7.50, *p* = 0.024, Friedman), with post hoc tests indicating changes from pre- to post- and pre- to follow-up (*p* = 0.016). The ANOVA revealed no significant effect in the remaining domains.

### Cognitive Task

At baseline, no significant group differences were found across the cognitive tasks, except in the FAS test, where the Sham group scored lower than the VLPFC group did (*p* = 0.016). All other comparisons were non-significant (*p* > 0.05), indicating comparable cognitive performance prior to the intervention. (Table A1)

1. TMT

A significant effect of time on the total time to complete the TMT was observed in the DLPFC group, as indicated by the Friedman test (χ²(2) = 7.00, *p* = 0.03, W = 0.44). Performance improved from pre- to post-intervention (adjusted *p* = 0.05) and from pre- to follow-up (adjusted *p* = 0.02), with no significant difference between post and follow-up (*p* = 0.54).

The VLPFC group showed a marginal effect in the same measure (χ²(2) = 5.60, *p* = 0.06, W = 0.28), suggesting a trend toward improvement over time.

In the Sham group, the Friedman test also revealed a significant effect of time (χ²(2) = 9.00, *p* = 0.01, W = 0.75), with significant improvements from pre- to post-intervention (adjusted *p* = 0.002), and from pre- to follow-up (adjusted *p* = 0.002), and no difference between post and follow-up.

2. CBTT

For this task, we analysed the total score, which was calculated as the product of the number of correct trials and the block span achieved in each condition. Since the data did not meet the assumption of normality, the Friedman test was used to assess changes over time. No significant differences in performance were observed across time points in any of the groups: the DLPFC (χ²(2) = 2.89, *p* = 0.24, Kendall’s W = 0.18), VLPFC (χ²(2) = 1.56, *p* = 0.45, W = 0.08), and Sham (F(2,10) = 0,37, *p* = 0.7, ηp²= 0.07). All effect sizes were small, indicating minimal or no change over time.

3. WCST

For this analysis, the Friedman test was used to assess changes over time in all groups, except for Total Errors in the Sham group, where the data met normality assumptions and a repeated-measures ANOVA was applied.

In the DLPFC group, no effect was found for Total Correct (χ²(2) = 5.24, *p* = 0.07, W = 0.33), but a significant effect was found for Total Errors (χ²(2) = 6.75, *p* = 0.03, W = 0.42), with reductions from pre- to post-intervention (*p* = 0.05) and from pre- to follow-up (*p* = 0.05). No changes were found between post and follow-up.

In the VLPFC group, no significant changes were detected in Total Correct (χ²(2) = 2.00, *p* = 0.37, W = 0.10), and only a marginal effect was detected for Total Errors (χ²(2) = 5.89, *p* = 0.05, W = 0.30), with trends toward improvement that did not survive correction (*p* > 0.05).

In the Sham group, no significant changes were found in Total Correct (χ²(2) = 3.82, *p* = 0.15, W = 0.32) and Total Errors (F(2,10) = 2.85, *p* = 0.11, ηp²= 0.36).

4. FAS

For the total score in the FAS task, changes over time were assessed using repeated-measures ANOVA in all groups. A significant effect of time was found in both the DLPFC (*F(2*,*14)* = 8.81, *p* = 0.003, ηp²= 0.55) and Sham (*F(2*,*10)* = 20.98, *p* < 0.001, ηp²=0.81) groups, with improvements from pre- to post-intervention and from pre- to follow-up (*p* = 0.02 and 0.02; 0.002 and 0.006, respectively), and no differences were detected between post and follow-up. The VLPFC group showed no significant effect over time (*F(2*,*18)* = 2.38, *p* = 0.12, ηp²= 0.21). A significant baseline difference was observed between Sham and VLPFC (*p* = 0.016).

5. ANT

For the ANT task, statistical analyses were conducted using repeated-measures ANOVA or Friedman tests, depending on data distribution and sphericity.

For the alerting network, repeated-measures ANOVA was used for all groups. A significant effect of time was found in the DLPFC (F(2,14) = 3.84, *p* = 0.04, ηp²=0.35) and Sham group (F(1.02,5,11) = 8.35, *p* = 0.03 ηp²= 0.63; Greenhouse–Geisser correction applied), but post hoc comparisons were not significant after Holm correction. The VLPFC group showed no effect (F(2,18) = 0.17, *p* = 0.84, ηp²= 0.02).

For the orienting network, the data were analysed using ANOVA for the DLPFC group and Friedman tests for the VLPFC and Sham groups. No significant changes over time were observed (DLPFC: F(2,14) = 0.96, *p* = 0.41, ηp²=0.12; VLPFC: χ²2 = 0.80, *p* = 0.67, W = 0.04; Sham: χ²2 = 2.33, *p* = 0.31, W = 0.19).

For the conflict network, the Friedman test was applied to the DLPFC group, VLPFC and Sham groups. Only the VLPFC group showed a significant effect of time (χ²2 = 7.20, *p* = 0.03, W = 0.36), with improvements from post-intervention to follow-up (*p* = 0.02). No effects were observed in the DLPFC group or Sham group.

6. Stroop

For the Colour condition of the Stroop task, the DLPFC group showed a significant effect of time (χ²(2) = 8.67, *p* = 0.01), with significant improvements from pre to post (*p* = 0.004) and pre to follow-up (*p* = 0.02). The VLPFC group, analysed using repeated-measures ANOVA, also showed a significant effect of time (F(2,18) = 7.21, *p* = 0.005), with post hoc comparisons indicating differences between pre and post (*p* = 0.01) and pre and follow-up (*p* = 0.05).

In the Word condition, the Sham group showed a significant effect over time (χ²(2) = 8.32, *p* = 0.02), with improvements from pre to post (*p* < 0.001) and pre to follow-up (*p* = 0.004).

For the Word-Colour condition, changes over time were assessed using repeated-measures ANOVA in all groups. A significant effect of time was found in the DLPFC group (F2,14) = 4.99, *p* = 0.02, ηp²=0.42), with improvements from pre- to post-intervention and from pre- to follow-up (*p* = 0.03), and no changes between post and follow-up (*p* = 0.67). The VLPFC group showed a marginal effect (F(2,18) = 3.53, *p* = 0.05, ηp²=0.28), with no significant post hoc differences. No effect was observed in the Sham group (F(2,8) = 0.69, *p* = 0.53, ηp²=0.15).

**Table 2:** Summary of statistical analyses for each cognitive task across groups (DLPFC, VLPFC, Sham) and time points (pre, post, follow-up)

	Pre		Post		Follow-up		Test	*p* value	Size effect	Post Hoc comparisons		
	Mean	SD	Mean	SD	Mean	SD		p<	Kendall/ηp²	Pre vs. Post	Pre vs. Follow-up	Post vs. Follow-up
*DLPFC*												
**TMT**												
TMT total time	166495,11	61075,29	130750,78	53781,74	122914,75	53233,87	Friedman	0,03	0,44	0,05*	0,02*	0,54
**CBTT**												
Total score	58,33	29,48	59,78	23,17	49,13	16,81	Friedman	0,24	0,18			
**WCST**												
Total nb of correct responses	33,11	6,90	37,22	1,20	35,75	1,75	Friedman	0,07	0,33			
Total nb of error	12,00	9,04	6,22	2,64	7,38	4,87	Friedman	0,03*	0,42	0,05*	0,05*	1,00
**FAS**												
Total	36,78	5,63	43,22	3,42	46,75	7,80	ANOVArm	0,003*	0,55	0,02*	0,02*	0,22
**ANT**												
Alerting effect	26,06	25,20	61,47	46,39	61,14	36,12	ANOVArm	0,04*	0,35	0,11	0,11	0,90
Orienting effect	14,81	17,78	13,24	15,50	23,72	15,52	ANOVArm	0,41	0,12			
Conflict effect	81,90	29,73	73,90	19,46	89,20	107,55	Friedman	0,20	0,20			
**Stroop**												
Word	101,22	18,33	108,33	17,72	105,63	16,93	ANOVArm	0,08	0,30			
Colour	64,78	11,71	70,11	11,54	68,25	12,14	Friedman	0,01*	0,54	0,004*	0,02*	0,28
Word-Colour	43,22	8,73	47,67	9,14	47,75	8,89	ANOVArm	0,02*	0,42	0,03*	0,03*	0,67
*VLPFC*												
**TMT**												
TMT total time	153183,50	40437,58	127858,60	39247,48	122297,40	38091,92	Friedman	0,06	0,28			
**CBTT**												
Total score	54,00	28,40	52,10	38,39	55,50	26,78	Friedman	0,45	0,08			
**WCST**												
Total nb of correct responses	33,10	7,87	34,30	4,11	36,50	2,88	Friedman	0,37	0,10			
Total nb of error	12,90	9,34	9,00	7,42	7,00	4,55	Friedman	0,05*	0,30	0,07	0,08	0,80
**FAS**												
Total	46,60	12,92	48,00	15,33	53,20	15,84	ANOVArm	0,12	0,21			
**ANT**												
Alerting effect	47,85	34,40	50,96	21,08	52,10	25,66	ANOVArm	0,84	0,02			
Orienting effect	22,13	25,37	25,14	22,22	30,87	14,18	Friedman	0,67	0,04			
Conflict effect	80,97	40,96	88,97	32,90	68,76	24,13	Friedman	0,03*	0,36	0,26	0,26	0,02*
**Stroop**												
Word	99,20	14,62	104,20	10,53	105,50	14,67	ANOVArm	0,13	0,20			
Colour	65,90	9,89	72,20	9,83	72,20	12,89	ANOVArm	0,005**	0,45	0,01*	0,05*	1,00
Word-Colour	42,50	9,24	47,20	9,88	47,50	10,34	ANOVArm	0,05*	0,28	0,15	0,15	0,88
*Sham*												
**TMT**												
TMT total time	134060,7	36232,7	96633,4	22088,8	99312,3	19508,3	Friedman	0,01*	0,75	0,002*	0,002*	1,00
**CBTT**												
Total score	61,6	28,1	67,6	18,7	64,5	32,2	ANOVArm	0,70	0,07			
**WCST**												
Total nb of correct responses	34,4	2,7	36,6	1,6	36,8	1,6	Friedman	0,15	0,32			
Total nb of error	8,7	7,0	7,6	3,6	4,7	3,6	ANOVArm	0,11	0,36			
**FAS**												
Total	32,0	8,5	41,3	7,9	39,5	8,6	ANOVArm	0,001**	0,81	0,002*	0,006*	0,39
**ANT**												
Alerting effect	17,1	22,6	37,1	21,6	54,4	26,6	ANOVArm	0,03*	0,63	0,10	0,10	0,10
Orienting effect	26,1	19,1	22,9	26,3	29,6	4,6	Friedman	0,31	0,19			
Conflict effect	74,5	26,5	75,4	20,5	78,0	24,9	Friedman	0,61	0,08			
**Stroop**												
Word	101,7	6,7	114,8	8,8	111,2	5,8	Friedman	0,02*	0,83	< 0,001*	0,004*	0,20
Colour	73,2	14,0	78,7	14,8	76,8	13,6	ANOVArm	0,53	0,15			
Word-Colour	48,7	16,4	51,2	14,2	47,6	12,8	ANOVArm	0,53	0,15			

The table reports the test statistics, adjusted *p*-values (Holm correction), and effect sizes (Kendall’s W for Friedman tests and partial η² for ANOVAs). Statistically significant comparisons are marked with * (*p* < 0.05) or ** (*p* < 0.01)

TMT, Trail Making Test; WCST, Wisconsin Card Sorting Test; ANT, Attentional network test; SD, Standard Deviation

### fNIRS Results

**Fig. 6:**
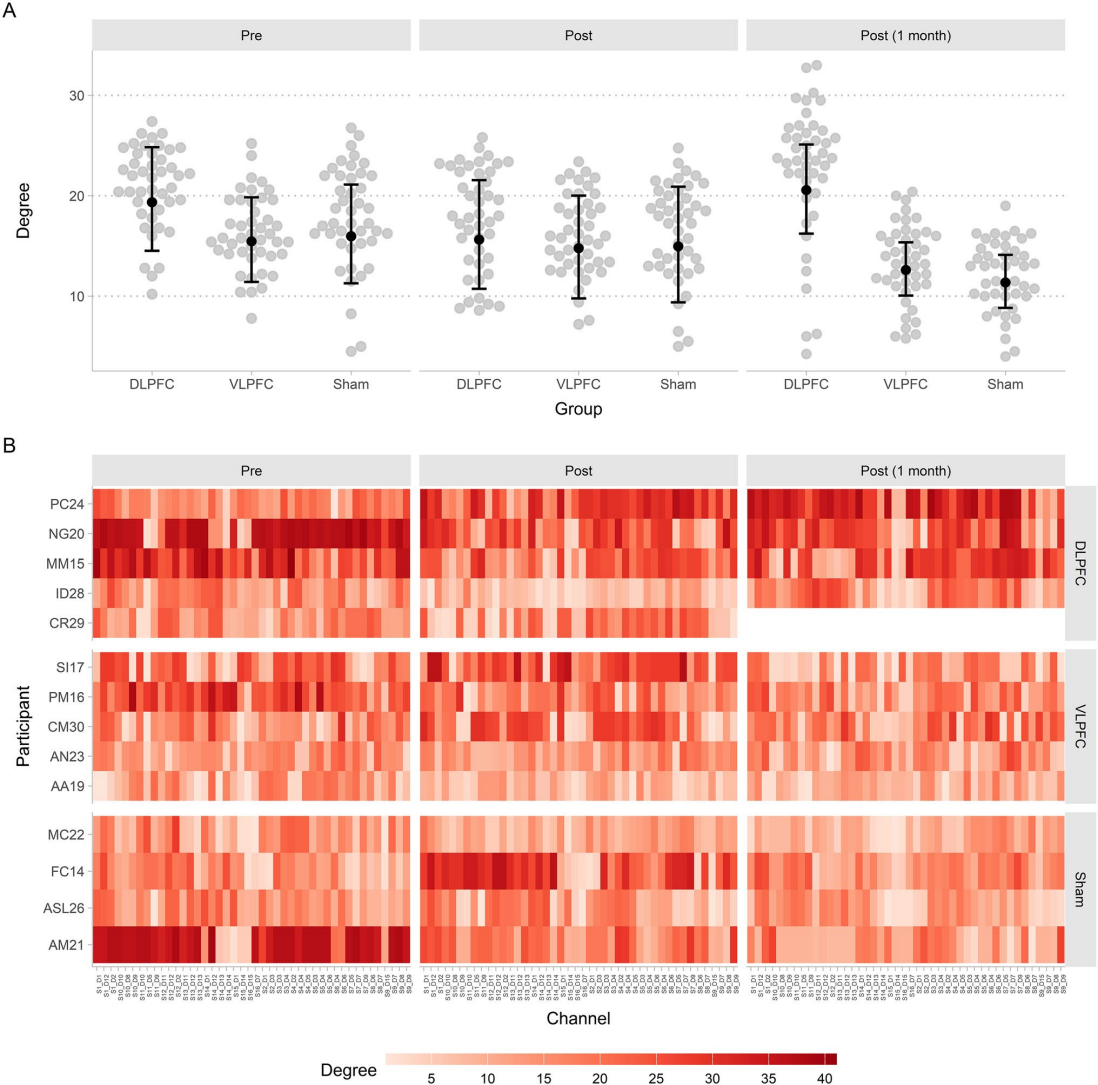
Average degree of connectivity by group, session, and fNIRS channel. (A) Average connectivity degree. Black dots and intervals indicate the mean and 89% HDI of the posterior predicted mean connectivity degree for each group in each recording session. Grey dots indicate the observed mean connectivity degree of each fNIRS channel. (B) Participant- and channel-wise connectivity degree across recording sessions

Model MCMC chains showed strong evidence of convergence ($\:\widehat{R}\le\:$ 1.01) and appropriate effective sample size (ESS $\:\ge\:$ 859), and simulation-based calibration checks revealed adequate model calibration (see Online Resource 1 for more details) (Modrák et al. 2025). Figure 6 shows the model-predicted degree of connectivity of each fNIRS channel across participants for each group and session.

1. Pairwise Comparisons by Group

**Fig. 7:**
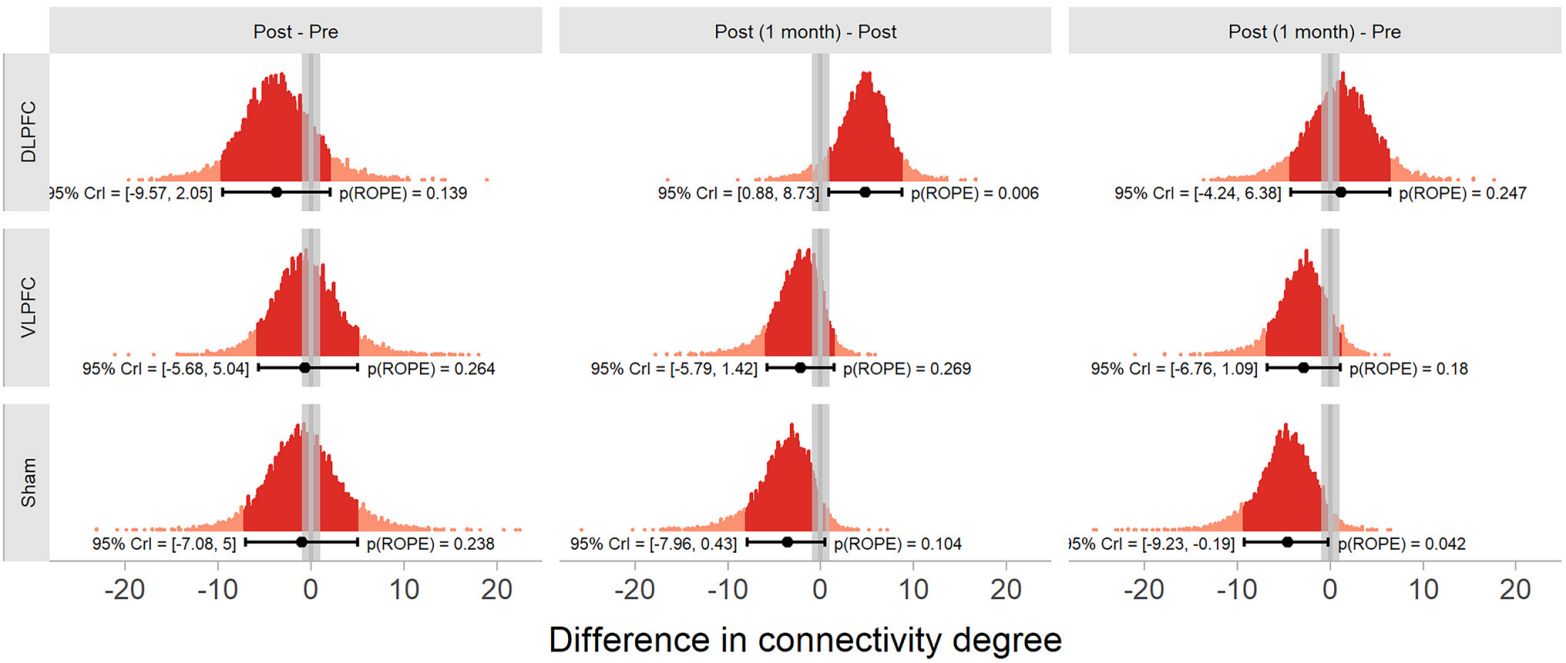
Posterior distribution of the pairwise differences between sessions for each group. Each dot in the distribution represents the difference between two posterior draws. Point intervals indicate the median and 89% HDI of the distribution. Grey rectangles indicate the ROPE

We first analysed the pairwise comparisons between each session, for each group separately (see Fig. 7). For participants in the sham group, the difference in degree of connectivity between before the HD-tDCS stimulation (Median = 15.97, 89% HDI = [11.28, 21.12]) and immediately after the HD-tDCS stimulation (Median = 14.96, 89% HDI = [9.38, 20.9]) included zero ($\:\varDelta\:$ = −0.95, 89% HDI = [−7.08, 5]) and 23.764% of the samples in its HDI overlapped with the ROPE. The difference in degree of connectivity between inmediately after the HD-tDCS stimulation and one month after the stimulation (Median = 11.37, 89% HDI = [11.28, 14.11]) included zero ($\:\varDelta\:$ = −3.57, 89% HDI = [−7.96, 0.43]) and 10.421% of the samples in its HDI overlapped with the ROPE. The difference in degree of connectivity between before the session and one month after the stimulation also included zero ($\:\varDelta\:$ = −4.6, 89% HDI = [−9.23, −0.19]) and 4.171% of the samples in its HDI overlapped with the ROPE.

For participants in the DLPFC group, the difference in degree of connectivity between before the HD-tDCS stimulation (Median = 19.35, 89% HDI = [14.52, 24.84]) and immediately after the HD-tDCS stimulation (Median = 15.64, 89% HDI = [10.73, 21.55]) included zero ($\:\varDelta\:$ = −3.69, 89% HDI = [−9.57, 2.05]) and 13.862% of the samples in its HDI overlapped with the ROPE. The difference in degree of connectivity between inmediately after the HD-tDCS stimulation and one month after the stimulation (Median = 20.57, 89% HDI = [14.52, 25.1]) included zero ($\:\varDelta\:$ = 4.8, 89% HDI = [0.88, 8.73]) and 0.590% of the samples in its HDI overlapped with the ROPE. The difference in degree of connectivity between before the session and one month after the stimulation also included zero ($\:\varDelta\:$ = 1.15, 89% HDI = [−4.24, 6.38]) and 24.691% of the samples in its HDI overlapped with the ROPE.

Finally, for participants in the VLPFC group, the difference in degree of connectivity between before the HD-tDCS stimulation (Median = 15.46, 89% HDI = [11.42, 19.85]) and immediately after the HD-tDCS stimulation (Median = 14.78, 89% HDI = [9.77, 20.01]) included zero ($\:\varDelta\:$ = −0.65, 89% HDI = [−5.68, 5.04]) and 26.447% of the samples in its HDI overlapped with the ROPE. The difference in degree of connectivity between inmediately after the HD-tDCS stimulation and one month after the stimulation (Median = 12.61, 89% HDI = [11.42, 15.36]) included zero ($\:\varDelta\:$ = −2.18, 89% HDI = [−5.79, 1.42]) and 26.938% of the samples in its HDI overlapped with the ROPE. The difference in degree of connectivity between before the session and one month after the stimulation also included zero ($\:\varDelta\:$ = −2.82, 89% HDI = [−6.76, 1.09]) and 18.034% of the samples in its HDI overlapped with the ROPE.

2. Pairwise Comparisons by Session

**Fig. 8:**
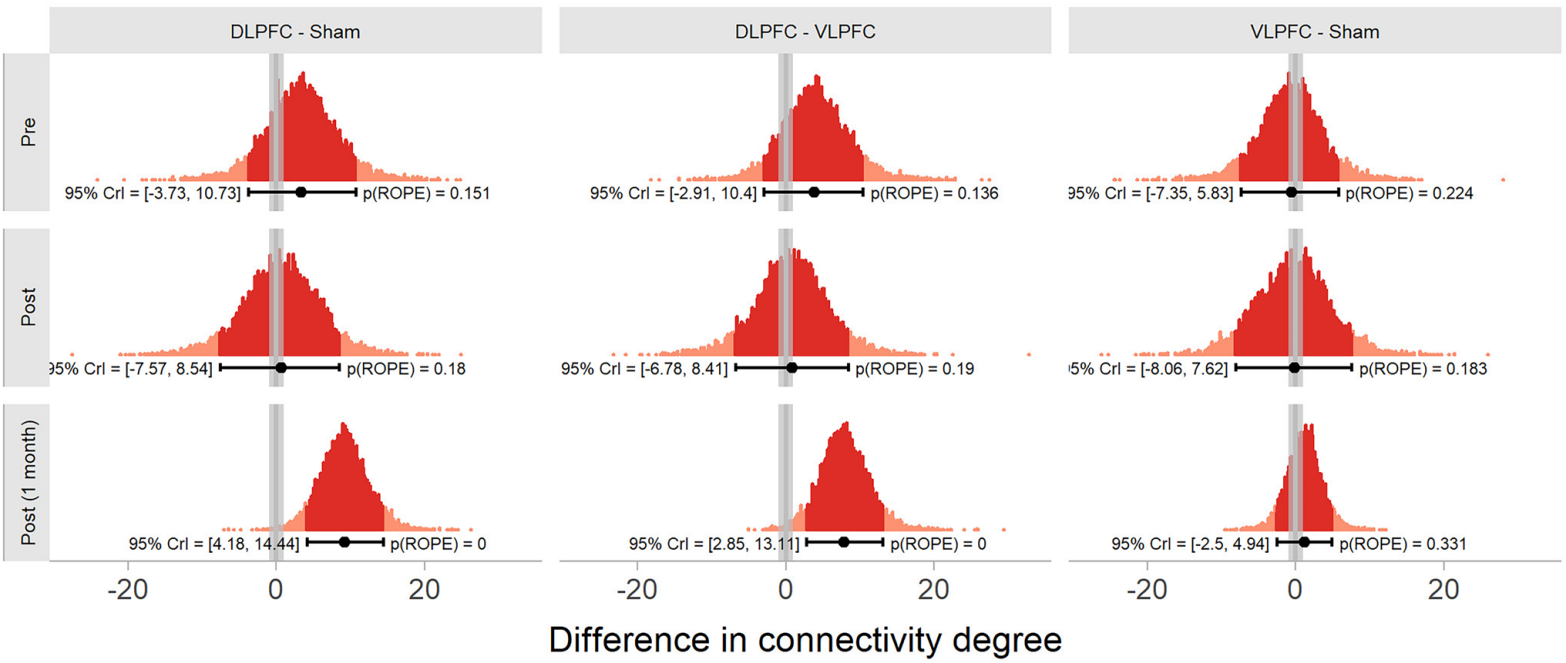
Posterior distribution of the pairwise differences between groups for each session. Each dot in the distribution represents the difference between two posterior draws. Point intervals indicate the median and 89% HDI of the distribution. Grey rectangles indicate the ROPE

We then analysed the pairwise comparisons between each group for each session separately (see Fig. 8). Before the HD-tDCS stimulation, the difference in degree of connectivity between participants in the DLPFC and sham groups included zero ($\:\varDelta\:$ = 3.38, 89% HDI = [−3.73, 10.73]) and 15.112% of the samples in its HDI overlapped with the ROPE. The difference in degree of connectivity between participants in the VLPFC and sham groups included zero ($\:\varDelta\:$ = −0.53, 89% HDI = [−7.35, 5.83]) and 22.388% of the samples in its HDI overlapped with the ROPE. The difference in degree of connectivity between participants in the VLPFC and sham groups included zero ($\:\varDelta\:$ = 3.87, 89% HDI = [−2.91, 10.4]) and 13.596% of the samples in its HDI overlapped with the ROPE.

Immediately after the HD-tDCS stimulation, the difference in degree of connectivity between participants in the DLPFC and sham groups included zero ($\:\varDelta\:$ = 0.73, 89% HDI = [−7.57, 8.54]) and 18.020% of the samples in its HDI overlapped with the ROPE. The difference in degree of connectivity between participants in the VLPFC and sham groups included zero ($\:\varDelta\:$ = −0.08, 89% HDI = [−8.06, 7.62]) and 18.329% of the samples in its HDI overlapped with the ROPE. The difference in degree of connectivity between participants in the VLPFC and sham groups included zero ($\:\varDelta\:$ = 0.88, 89% HDI = [−6.78, 8.41]) and of the samples in its HDI overlapped with the ROPE.

Finally, one month after the HD-tDCS stimulation, the difference in degree of connectivity between participants in the DLPFC and sham groups excluded zero ($\:\varDelta\:$ = 9.17, 89% HDI = [4.18, 14.44]) and none of the samples in its HDI overlapped with the ROPE. The difference in degree of connectivity between participants in the VLPFC and sham groups included zero ($\:\varDelta\:$ = 1.26, 89% HDI = [−2.5, 4.94]) and 33.132% of the samples in its HDI overlapped with the ROPE. The difference in degree of connectivity between participants in the VLPFC and sham groups excluded zero ($\:\varDelta\:$ = 7.89, 89% HDI = [2.85, 13.11]) and none of the samples in its HDI overlapped with the ROPE.

## Discussion

The present study investigated whether administering 10 days of HD-tDCS to either the DLPFC or VLPFC would produce distinct effects on depressive symptoms, quality of life, and cognitive control in individuals with MDD. By combining self-report measures of depression and quality of life with cognitive tasks and resting-state fNIRS, this study aimed to capture changes in symptomatology, performance, and neural activity. This approach was designed to elucidate the specific roles of these prefrontal regions and evaluate their potential as neuromodulation targets for the cognitive deficits associated with depression.

### Depression Assessment

Results from the BDI questionnaire revealed distinct patterns of improvement across groups. Participants receiving DLPFC stimulation showed a persistent reduction in depressive symptoms, with significant decreases observed both immediately after the intervention and at the one-month follow-up. These findings suggests not only an acute but also a lasting effect of stimulation over this prefrontal target, which is consistent with previous findings highlighting the DLPFC’s central role in mood regulation (Salehinejad et al. 2017; Wong et al. 2019; Asgharian Asl and Vaghef 2022; Ngan et al. 2022). In contrast, the VLPFC group showed a more gradual response, with significant improvements emerging only at the one-month follow-up. This delayed effect may reflect a slower engagement of emotional regulation networks associated with VLPFC stimulation or perhaps a more cumulative neuroplastic process (Ochsner et al. 2012; Silvers et al. 2017; He et al. 2023). In comparison, the Sham group did not show significant improvements over time, indicating that the observed therapeutic benefits under active conditions are more likely attributable to the neuromodulatory effects of HD-tDCS.

### Quality of Life

In terms of quality of life, stimulation over the DLPFC led to notable improvements, particularly in the psychological and physical health domains. Although changes in the total score were only marginally significant, the consistent trend across time points supports the potential of DLPFC-targeted HD-tDCS to enhance well-being in individuals with depression. These findings align with the established role of the DLPFC in emotional and self-regulatory processes (Ochsner et al. 2004; Golkar et al. 2012). In contrast, the VLPFC group did not exhibit significant changes, suggesting a more limited impact on broader quality of life measures. Unexpectedly, the Sham group showed improvements in physical health, which could reflect nonspecific or placebo-related effects. Additionally, it is possible that requiring attendance at the university daily for 10 consecutive sessions improved physical health by encouraging participants to leave the house and regularly be active, independent of the stimulation received. The lack of improvement in the VLPFC group, despite the same behavioural demands, may be due to the subjective effects of active stimulation, such as mild discomfort or headache, which could have blunted the perceived benefits of daily engagement. However, this pattern is unexpected and difficult to interpret, highlighting the complexity of disentangling specific and nonspecific effects in neuromodulation studies.

### Cognitive Performance

Beyond depression and quality of life assessment, we explored whether HD-tDCS had an impact on cognitive performance. The following section focuses on results from tasks assessing cognitive functions.

Performance improvements in the TMT were observed following DLPFC stimulation, with significant reductions in completion time from pre- to post-intervention and sustained at follow-up. These findings align with previous studies showing that tDCS targeting the DLPFC can enhance cognitive control and processing speed, two core components assessed by the TMT (Hunter et al. 2015). Notably, the Sham group also exhibited significant gains, likely driven by practice effects, which are well documented in repeated administrations of the TMT (Bartels et al. 2010). The VLPFC group showed only a marginal improvement, possibly due to limited power to detect small effects and, similarly, was influenced by task repetition. Given this, it is also possible that the improvements observed in the DLPFC group reflect, at least in part, these same practice-related effects rather than a specific impact of stimulation. This interpretation is supported by Yildiz et al., (2023), who reported significant TMT improvements in both the sham and active stimulation groups after 20 sessions of rTMS over the left DLPFC, suggesting that the effects may be attributable to practise-related mechanisms.

Consistent with previous studies in other clinical populations, such as those with ADHD (Guimarães et al. 2024), we observed no significant improvement in the CBTT following HD-tDCS stimulation. The CBTT’s high variability and moderate reliability may hinder the detection of small changes (de Paula et al. 2016). This suggests that, at higher levels of performance, the task may fail to detect subtle differences, reflecting a limitation commonly attributed to ceiling effects (García-Sevilla et al. 2014).

Participants who received stimulation over the DLPFC showed a significant reduction in the number of errors in the WCST, both immediately after the intervention and at follow-up. This suggests an improvement in cognitive flexibility and error-monitoring processes. However, drawing on the work of Mansouri et al. (2007, 2016), it is important to note that the DLPFC may not directly influence the initial number of errors or conflict cost per se, but rather plays a key role in error-induced behavioural adaptation. Their studies in both macaque monkeys and humans with DLPFC lesions, as well as in healthy participants receiving tDCS, show that this region is crucial for adjusting behaviour after conflict or error, rather than for avoiding errors from the outset. In line with this, the reduction in errors observed in our study may reflect improved post-error adjustment and set-shifting over time, rather than immediate conflict resolution. This interpretation is further supported by findings that cathodal tDCS over the DLPFC can modulate post-error slowing, specifically under high-conflict conditions (Mansouri et al. 2016). Thus, the effect observed here may be better explained by the stimulation helping participants improve how they adapt and adjust their behaviour during executive tasks. In contrast, no such effect was observed in the Sham group, supporting the specificity of the effect. The VLPFC group showed an improvement that did not survive correction, suggesting a limited role of this region in the adaptive components of the WCST.

Performance on the FAS task improved significantly over time in both the DLPFC and Sham groups, but not in the VLPFC group. While these improvements in the DLPFC group might reflect stimulation-related effects on verbal fluency supported by this region, the similar pattern observed in the Sham group suggests that practice effects played a substantial role. This finding is consistent with previous findings indicating that verbal fluency tasks are particularly susceptible to learning across repeated assessments (Jin et al. 2023). Moreover, studies such as Moreno et al. (2020) reported “reduced practice gains” in tDCS-treated patients compared to sham, highlighting the complex interaction between stimulation and task repetition. In our data, the absence of significant change in the VLPFC group may partly reflect a ceiling effect, as this group showed higher baseline performance compared to Sham, potentially leaving less room for measurable improvement.

In the ANT task, no significant effects were found in any group for the alerting and orienting networks, which is consistent with previous studies reporting low reliability for these components (Mash et al. 2020; Kong et al. 2024). In contrast, the conflict network showed significant post to follow-up improvement in the VLPFC group but not in the other two groups, indicating better conflict resolution. This may reflect the modulation of inhibitory processes and increased efficiency in filtering distractions. The VLPFC, as a hub of the ventral attention and salience networks (Corbetta and Shulman 2002; Trambaiolli et al. 2022), supports attentional reorientation and the integration of relevant stimuli. While its role differs from the DLPFC’s more direct involvement in conflict resolution (Liu et al. 2023), the VLPFC contributes through inhibition, reorientation, and network coordination (Morawetz et al. 2017; Trambaiolli et al. 2022). It is also involved in emotional regulation and executive inhibition (Goto et al. 2011; Chao et al. 2021; He et al. 2023), with studies linking its dysfunction to attentional biases in depression (Morawetz et al. 2017; Marques et al. 2018; Liu et al. 2019). Thus, delayed conflict improvement may reflect enhanced flexibility and emotional control. Together, these findings suggest that VLPFC stimulation facilitates conflict adaptation over time, supporting functional dissociation between prefrontal subregions in attentional regulation.

In the Stroop Colour-Word condition, only the DLPFC group showed significant improvement over time, with gains from pre- to post-intervention sustained at follow-up. This aligns with prior findings showing that DLPFC stimulation enhances performance in interference-based tasks like the Stroop test (Concerto et al. 2015; Yildiz et al., 2023), often through reduced reaction times (Wadsley 2019) and improved overall accuracy (Concerto et al. 2015; Yildiz et al., 2023). These effects are linked to the executive control network, which involves the DLPFC and anterior cingulate cortex (Streeter et al. 2008; Wadsley 2019). The persistence of these improvements suggests that neuromodulation over the DLPFC may promote not only short-term gains in inhibitory control but also longer-lasting cognitive effects, especially in depression, where such deficits are well documented (Epp et al. 2012). In contrast, the VLPFC group showed no significant post hoc effects, and the Sham group showed no effects, reinforcing the specificity of the DLPFC response. Slight improvements in simpler word and colour conditions across all groups likely reflect practice effects, highlighting the importance of interpreting high-conflict trials separately (Davidson et al. 2003).

Overall, our results suggest that some improvements (e.g., in the Trail Making Test, verbal fluency, or Word Stroop) are best explained by practice or nonspecific effects, as they were also observed in the sham group. In contrast, the more selective gains in Stroop performance (DLPFC group), WCST error reduction (DLPFC group), and conflict monitoring in the ANT (VLPFC group) are more likely attributable to stimulation-driven effects.

Beyond this distinction, our results also demonstrate a functional dissociation between the DLPFC and VLPFC in how they modulate depressive symptoms and cognitive control. Stimulation over the DLPFC was associated with improvements that emerged shortly after the intervention and were observed across several cognitive domains, including flexibility, inhibition, and verbal fluency, which is consistent with the role of the DLPFC in top-down regulation, goal maintenance, and adaptive behaviour (Wagner et al. 2001; Goto et al. 2011; Chan and Han 2020; Zheng et al. 2023). In contrast, stimulation over the VLPFC was associated with more delayed and selective improvements, primarily observed at follow-up in tasks involving conflict resolution and interference control, which aligns with the proposed role of this region in inhibitory processing and attentional reorientation (Goto et al. 2011; He et al. 2023). These findings suggest that neuromodulation strategies should be adapted to the distinct roles of each prefrontal region, depending on the specific cognitive or emotional processes we expect to impact.

### Neuromodulation

Neuromodulation techniques, including tDCS, transcranial alternating current stimulation (tACS), transcranial random noise stimulation (tRNS), and focused ultrasound stimulation (FUS), offer promising approaches for the treatment of MDD. Each method differs in the type of energy delivered, its primary mechanism, and spatial precision. For instance, tACS uses alternating current to entrain endogenous oscillations and has shown potential to modulate mood and cognition by targeting specific frequencies (Yao et al. 2025). tRNS delivers random noise within a frequency band, potentially enhancing cortical excitability through stochastic resonance; however, clinical evidence in MDD remains limited, and results are inconsistent (Schecklmann et al. 2021). FUS, particularly low-intensity FUS (LIFUS), provides unique submillimeter precision and the ability to noninvasively reach deep brain regions, with early studies suggesting clinical promise but still in an experimental stage (Folloni et al. 2019).

In contrast, tDCS is distinguished by its accessibility, safety, and a substantially broader evidence base, particularly in depression. Anodal stimulation over the left DLPFC has been shown to reduce depressive symptoms and improve cognitive control, and meta-analyses confirm its clinical efficacy compared to sham (Brunoni et al. 2017). Importantly, tDCS is well tolerated, cost-effective, and can be combined with pharmacological or psychological interventions, which significantly enhances treatment outcomes (Wang et al. 2021). Moreover, recent work demonstrates that its neurophysiological effects can persist beyond the stimulation period, for example in working memory–related alpha activity (Murphy et al. 2023). While newer modalities, such as FUS or tACS, hold considerable potential, tDCS currently remains one of the most clinically relevant neuromodulation tools due to its balance of efficacy, safety, and feasibility for large-scale applications.

### Functional Connectivity

In addition to behavioural and clinical outcomes, fNIRS was used to investigate whether HD-tDCS led to changes in brain connectivity in the resting state. Functional connectivity was quantified using the degree of each channel within a binarized correlation matrix.

tDCS is thought to modulate functional connectivity by inducing sustained changes in cortical excitability and synaptic plasticity. Anodal stimulation has been shown to reduce local GABAergic inhibition (Bachtiar et al. 2015), thereby facilitating network-level reorganization and increasing resting-state functional connectivity(Polanía et al. 2011). Beyond local effects, tDCS generates electric fields that influence large-scale prefrontal networks (Yaqub et al. 2018), and repeated sessions are believed to promote long-term potentiation (LTP)-like plasticity (Kuo et al. 2013). Such processes may strengthen prefrontal–parietal circuits involved in executive control and reduce maladaptive prefrontal–limbic coupling associated with depressive symptoms. These network-level effects are consistent with prior work using graph-theoretical approaches to show that tDCS alters topological organization of brain networks at rest (Polanía et al. 2011).

Contrary to initial expectations, comparisons within each stimulation group across sessions did not reveal conclusive evidence of change in overall connectivity degree. For the DLPFC group, although a slight increase was observed from post-intervention to the one-month follow-up, the 89% HDI of the difference in connectivity degree between sessions included zero and overlapped with the ROPE, indicating that observed differences between sessions cannot be confidently considered different than zero, given the data. Similarly, no significant within-group changes were observed in the VLPFC or sham groups across the time points. These results suggest that repeated HD-tDCS may not induce consistent or immediate modulation of global connectivity density in the frontal cortex.

However, the spatial distribution of functional connections, as illustrated in Online Resource 2, provides additional qualitative insight into possible stimulation effects. At the post-stimulation session, the DLPFC group exhibited a noticeable shift in connectivity patterns, with above-threshold links becoming more concentrated within anterior prefrontal regions rather than being broadly distributed across the frontal cortex, as observed at baseline. This suggests that stimulation may have promoted a reconfiguration of the functional network architecture, altering where connections are expressed rather than how many are formed. While exploratory in nature, this topographical reorganisation may reflect a more efficient or functionally targeted integration of prefrontal circuits, potentially underlying the behavioural and clinical effects observed. These findings align with previous studies showing that tDCS can alter the topological organisation of brain networks. Polanía et al. (2011) reported polarity-specific changes in graph metrics, suggesting modulation of large-scale network configurations. Extending this to clinical populations, Sergiou et al. (2023) showed that prefrontal stimulation altered connectivity patterns in violent offenders with substance dependence. In this study, both HD-tDCS and electroencephalography (EEG) recordings were employed to stimulate the brain and measure neural activity, respectively. Notably, despite these changes in network organisation, no effects were found on spectral power in EEG recordings, suggesting that HD-tDCS may affect how brain networks are structured rather than changing the overall strength of neural activity.

A different pattern emerged when comparing groups at the follow-up session. One month after the intervention, participants who had received stimulation over the DLPFC exhibited a significantly higher degree of connectivity than did those in the sham group, with no overlap with the ROPE. Furthermore, the DLPFC group also presented significantly greater connectivity than did the VLPFC group. These differences between groups, despite the absence of within-group changes, suggest that HD-tDCS over the DLPFC may alter the long-term trajectory of functional network organisation in a way that diverges from natural or placebo-related fluctuations. The observed greater difference in resting-state functional connectivity in the DLPFC group than in the other groups at the one-month follow-up suggested that the effects of HD-tDCS are not transient but may reflect durable neuroplastic changes. Such long-lasting modulation of prefrontal connectivity may underlie sustained improvements in cognitive functions, such as cognitive flexibility, inhibition, and verbal fluency. Moreover, enhanced functional organization of prefrontal networks may contribute to greater cognitive resilience, potentially reducing the risk of relapse in future depressive episodes. This interpretation is supported by previous evidence showing that individuals with better cognitive control performance and more efficient cognitive control network (CCN) activation and connectivity are less likely to experience MDD recurrence, whereas disrupted CCN-Salience and Emotional Network interactions have been identified as markers of increased vulnerability (Langenecker et al. 2018).

This finding aligns with the hypothesized role of the DLPFC as a hub in frontoparietal control networks, which are capable of exerting a top-down influence on distributed cortical systems (Miller and Cohen 2001; Goto et al. 2011; Patel et al. 2024) Increased connectivity degree in this region may reflect enhanced neural integration or efficiency, potentially supporting the behavioural improvements observed in executive function and mood (Mancini et al. 2016; Jog et al. 2021). It is also notable that these effects were present only at follow-up, not immediately after the intervention, which suggests that the neuromodulatory effects of HD-tDCS may require time to consolidate, possibly via synaptic plasticity or delayed neurovascular changes (Liebetanz et al. 2002; Hunter et al. 2015; Jog et al. 2021).

In contrast, stimulation over the VLPFC did not lead to significant changes in resting-state connectivity when compared to sham. This may reflect a more functionally specific or transient role of the VLPFC in cognitive and affective processing, with neural effects that are not captured by global measures of connectivity in the resting state (Wagner et al. 2001) Alternatively, the VLPFC may engage subcortical or posterior circuits like the Nucleus Accumbens or Insula, that lie outside the coverage area of fNIRS montage. (Luna et al. 2010; Nguyen et al. 2018; Vassena et al. 2019; He et al. 2023). According to Barbas’ structural model of cortical connectivity, the VLPFC tends to send projections primarily to the deeper layers of its target regions, particularly layer V, which are involved in output to subcortical structures and motor systems (Barbas and Rempel-Clower 1997; Barbas 2015). This laminar projection pattern suggests that the VLPFC may exert its influence through more unidirectional or subcortically mediated pathways, rather than through widespread cortico-cortical integration, potentially limiting the detectability of its effects in resting-state connectivity analyses.

fNIRS results suggest that HD-tDCS over the DLPFC may induce delayed, spatially specific changes in brain connectivity, evident one month after the intervention. While no consistent within-group effects were observed, increased connectivity at follow-up compared to sham and VLPFC supports a potential long-term modulation of network organisation. These effects may reflect gradual consolidation processes rather than immediate changes. In contrast, stimulation over the VLPFC had no clear impact, possibly due to its more localized function or limitations in fNIRS spatial coverage.

### Limitations

Several limitations of the present study should be acknowledged. The most important limitation is the relatively small sample size, especially considering the division into three stimulation groups. This inevitably limits the statistical power to detect subtle effects and increases the likelihood of false negatives and overestimation of true effect sizes. Although post-hoc sensitivity analyses (G*Power) confirmed that the design was adequately sensitive to detect the medium-to-large effects observed in key cognitive tasks, it was likely underpowered to reliably capture more subtle changes. In addition, not all outcomes allowed for formal power estimation: tasks analysed with Friedman tests (reported with Kendall’s W) and clinical questionnaires (BDI, WHOQOL-BREF) should therefore be interpreted with caution. Future studies involving larger and more diverse clinical populations will be essential to strengthen the robustness and generalizability of these findings.

Although a sham condition was included to control for expectancy effects, the study was not double-blinded. This leaves open the possibility of subtle experimenter bias during data collection or participant interaction, even if participants themselves were unaware of their group assignment.

The one-month follow-up can already be considered a marker of longer-term effects compared to immediate post-intervention outcomes, extending the follow-up to six months or even one year would provide a more complete picture of the stability and potential clinical relevance of the observed changes.

Important limitations of fNIRS should be acknowledged when interpreting functional connectivity results. First, the technique is restricted to superficial cortical regions due to limited depth penetration of near-infrared light, and thus cannot capture subcortical or deeper cortical contributions (Pinti et al. 2020; Sui et al. 2022). Second, fNIRS signals are sensitive to extracerebral hemodynamics, such as scalp blood flow, which can contaminate measures of connectivity. The use of short-separation channels can help mitigate these systemic artifacts (Yaqub et al. 2018). Motion-related artifacts remain another challenge (Brigadoi et al. 2014), although several correction methods, such as TDDR, have been developed (Fishburn et al. 2019; Hou et al. 2021). Finally, reproducibility is affected by heterogeneity in preprocessing pipelines and ROI selection strategies, which complicates comparison across studies(Yücel et al. 2021). These considerations suggest that interpretations should remain cautions. Future studies will benefit from multimodal designs (e.g., fMRI–fNIRS, EEG–fNIRS) and the adoption of transparent, standardized preprocessing pipelines to strengthen confidence in connectivity-based biomarkers of stimulation effects, and larger samples will be required to improve generalizability. In addition, the degree metric, while informative, does not capture directionality, connection strength, or modular network structure.

Another consideration is the the use of a 10-day stimulation protocol. This choice was guided by previous clinical and cognitive studies showing that this protocol is effective in reducing depressive symptoms (Boggio et al. 2008; Rigonatti et al. 2008; Loo et al. 2010; Wong et al. 2019; Moirand et al. 2022; Ngan et al. 2022) and in improving executive functions when targeting the DLPFC (Vanderhasselt and De Raedt 2009; Imperio and Chua 2023; Lu et al. 2025). Benefits of 10 sessions have also been reported to persist for up to three months (Boggio et al. 2008; Wong et al. 2019). Nevertheless, the overall efficacy of neuromodulation is considered dose-dependent, and longer courses (20 + sessions) may yield more robust or sustained effects(Ghazi-Noori et al. 2024). However, large randomized controlled trials with extended protocols have produced mixed findings, with some failing to show superiority of active over sham (Loo et al. 2012) or reporting antidepressant medication to be more effective than tDCS (Brunoni et al. 2017). Future research should therefore compare different dosing regimens and include longer follow-up periods to establish the optimal duration for lasting clinical and cognitive benefits. Future studies should therefore include larger and more diverse samples to increase sensitivity and enhance the generalisability of these findings.

Finally, while these results do not offer definitive proof of neuroplasticity, they suggest diverging post-stimulation trajectories that should be explored in further research.

## Conclusion

This study provides preliminary evidence that repeated HD-tDCS over the DLPFC and VLPFC can differentially modulate cognitive and emotional functioning, as well as quality of life, in individuals with MDD. DLPFC stimulation was associated with earlier and broader improvements in mood, quality of life, and cognitive performance, whereas VLPFC effects appeared more selective and delayed, particularly in conflict adaptation. Although no consistent within-group changes were observed in resting-state connectivity, spatial reorganisation of functional networks and between-group differences at follow-up suggest lasting modulation of prefrontal connectivity, especially in the DLPFC. These findings support a functional dissociation between prefrontal subregions and have potential implications for the design of neuromodulation protocols aimed at improving cognitive and affective functioning in depression. Future studies with larger samples and longer follow-up periods will be essential to confirm and extend these results.

## Supplementary Information

Below is the link to the electronic supplementary material.

Supplementary Material 1

## Data Availability

Data and materials for this research are available on the Open Science Framework (https://osf.io/4bs3p/?view_only=eb8e007261fb42419d7144d3a9a0dde8), and code for fNIRS analysis is available on GitHub (https://github.com/gongcastro/tdcs-nirs-connectivity).

## Footnotes

1 Before entering the model, the *Group* and *Session* predictors were sum-coded, to facilitate the interpretation of the regression coefficients (Schad et al. 2020). Levels in the *Group* predictor were coded into two contrasts: one comparing the DLPFC and the VLPFC group (DLPFC = + 0.5, VLPFC = -0.5, Sham = 0), and one comparing the gran average of the DLPFC and VLPFC groups with the sham group (DLPFC = + 0.25, VLPFC = + 0.25, Sham = -0.5). Levels in the *Session* predictor were also coded into two contrasts: one comparing pre- and post-stimulation sessions (Pre = -0.5, Post = 0.5, Post (1 month) = 0), one comparing the two post-stimulation sessions (Pre = 0, Post = -0.5, Post (1 month) = + 0.5).
