# Supplementary material for: Dissociating the Role of Dorsolateral Prefrontal Cortex and Ventrolateral Prefrontal Cortex in Cognitive Control in Depression: A Combined HD-tDCS and fNIRS Study
Source: Brain Topogr. 2025 Nov 26;39(1):2. doi: 10.1007/s10548-025-01157-4 (PMC12657566; doi:10.1007/s10548-025-01157-4)
Supplement: Supplementary file 1 — Supplementary Material 1 [file 10548_2025_1157_MOESM1_ESM.docx]

Dissociating the Role of the dlPFC and vlPFC in Cognitive Control in Depression: A Combined HD-tDCS and fNIRS Study

A. Hernández-Sauret^1^, G. Garcia-Castro^2^, D.E. Redolar-Ripoll^1^

^1^ Cognitive Neurolab, Faculty of Health Sciences, Universitat Oberta de Catalunya (UOC), Rambla del Poblenou 156, Barcelona, Spain

^2^ NeuroDevelopment and Comparative Cognition, Institut de Recerca Sant Joan de Déu (IRSJD), Calle Santa Rosa 39-57, Esplugues de Llobregat, Spain

Corresponding author:

Mail: [anahsauret@uoc.edu](mailto:anahsauret@uoc.edu)

Telephone number: +34 648100999

A. Hernández-Sauret ORCID: 0000-0003-4738-8308

G. García-Castro ORCID: 0000-0002-8553-4209

D.E. Redolar-Ripoll ORCID: 0000-0001-6922-2822

**SUPPLEMENTARY MATERIAL**

**Model diagnostics**


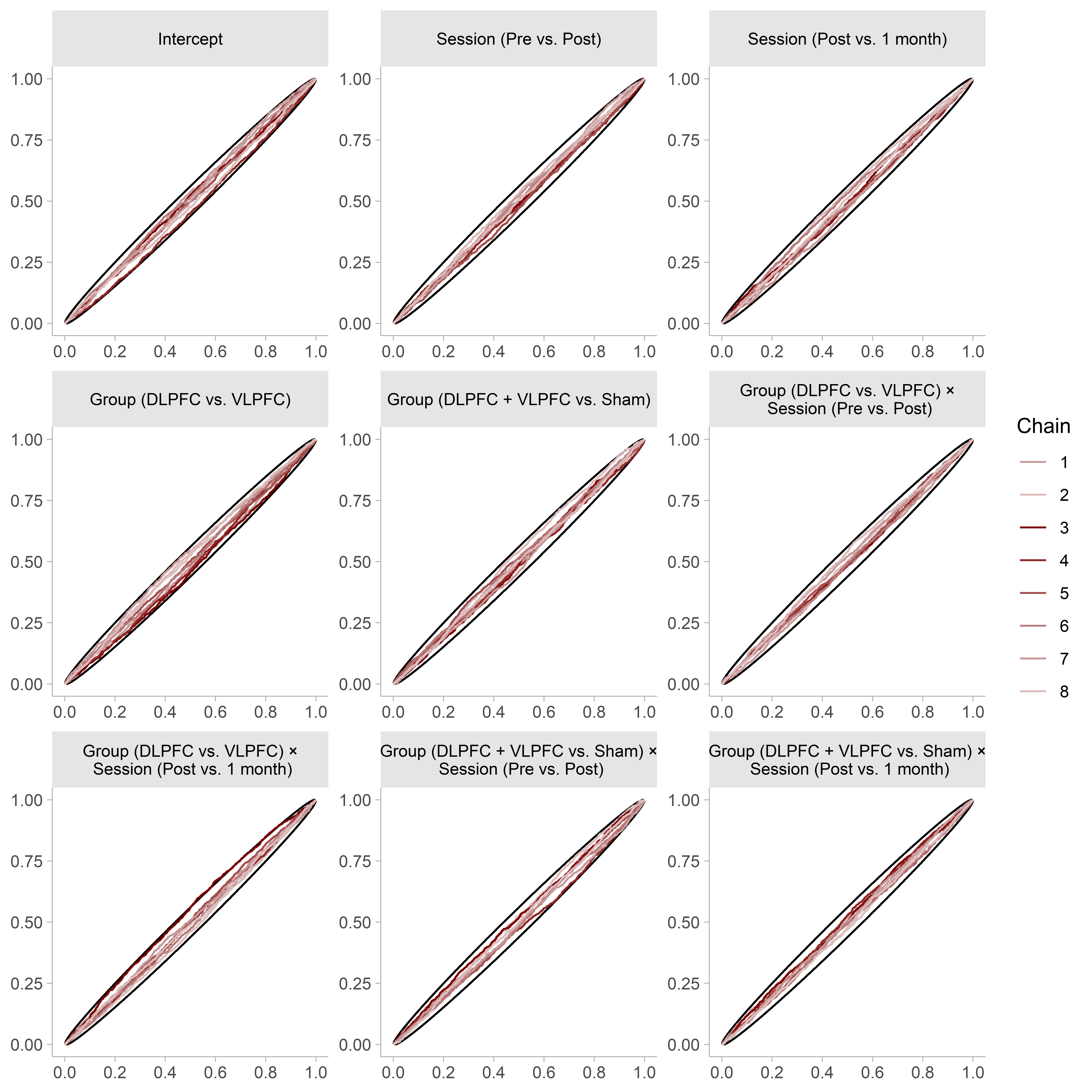


**Online Resource 1**. Empirical cumulative distribution function (ECDF) of the true-value posterior quantiles of the fixed regression coefficients of the model. Under good calibration, the ECDF of each chain (red) is expected to lie within the 99% confidence envelope (black).

**Connectivity circle**

|   **Online Resource 2**. Average connectivity circle for each group and session. Lines indicate above-threshold degree of connectivity. Channels are displayed in a circular fashion, with channels in the left (darker grey) and right (lighter grey) hemispheres on the corresponding side of the circle, and with more anterior channels on the top and more posterior channels on the bottom. |
| --- |
